# Catalytic liquefaction of sewage sludge to small molecular weight chemicals

**DOI:** 10.1038/s41598-020-75980-z

**Published:** 2020-11-03

**Authors:** Yuehu Wang, Feihong Tian, Peimei Guo, Dazhen Fu, Hero Jan Heeres, Taotao Tang, Huayu Yuan, Bing Wang, Jiang Li

**Affiliations:** 1 College of Resources and Environmental Engineering, Guizhou University, Guiyang, 550025 China; 2 Observation and Research Station for Guizhou Karst Environmental Ecosystems, Guiyang, 550025 China; 3 Chemical Engineering Department, ENTEG, University of Groningen, Nijenborg 4, 9747 AG Groningen, The Netherlands

**Keywords:** Environmental sciences, Chemistry

## Abstract

The catalytic hydrotreatment of sewage sludge, the wet solid byproducts from wastewater treatment plants, using supported Ir, Pt, Pd, Ru catalysts had been investigated with different solvent conditions. Reactions were carried out in a batch set-up at elevated temperatures (400 °C) using a hydrogen donor (formic acid (FA) in isopropanol (IPA) or hydrogen gas), with sewage sludge obtained from different sampling places. Sewage sludge conversions of up to 83.72% were achieved using Pt/C, whereas the performance for the others catalysts is different and solvent had a strong effect on the conversion rate and product constitution. The sewage sludge oils were characterised using a range of analytical techniques (GC, GC–MS, GCxGC, GPC) and were shown to consist of monomers, mainly alkanes and higher oligomers.

## Introduction

Sewage sludge includes dewatered sewage of wastewater treatment plant, digging sludge of city sewer and sum wadi dirt mud^1^. Dewatered sewage from sewage process factory is the main source. Dewatered sewage of sewage process factory is the half solid or solid material in sewage process course, and uninclude slag, scum, and grit. Usually quantity of dewatered sewage increases with the increace of sewage output amount and process quantity due to the acceleration of urbanization. For example, in china, by september 2016, there were 3976 sewage process factories, and capacity of sewage process is about 170 million m^3^/day. Amount of wet dewatered sludge of 80% moisture content reached to 45,900 thousand ton per year if the produce rate of dewatered sludge refer to 1.5 ton sewage sludge per ten thousand ton waste water^2^. Dewatered sewage sludge will attain to 60,000–90,000 thousand ton to 2020 depend on the forecast of correlated nation department of china^3^. The harmless treatment disposal rate of dewatered sewage sludge is only 33% and 67% was disposed without harmless treatment disposal. Random stack of dewatered sewage sludge has led to serious environment pollution and bring to a series of economy and society problems.

On the one hand, sewage sludge is the heterogeneous mixture what was proceed and aggregated in all sedimentation tank, which is composed of organic relic, microorganism causative agent, parasite egg, inorganic granule, and colloid. It is putrescibility and metamorphism, and then foul maleficence gas will be distributed if it is not processed in time and cause to severe environment pollution, and bring sever health menace to human. Its main characteristic is moisture content is very high (95%–99%), dewater of it is difficult and its volume is usually huge, which make its process and handle is difficult and transport expense is considerable. On the other hand, dewatered sewage sludge not only include abundant organic carbon source, moreover it also contains mass heavy metal as well as abundant nitrogen, phosphorus and kalium, so it could be recycled as a sort of resource. Vinay Kumar Tyagi and others suggested dewatered sewage sludge could be used as a reproducible resource of energy and organic chemical as early as 2013^4^.

**Figure 1:**
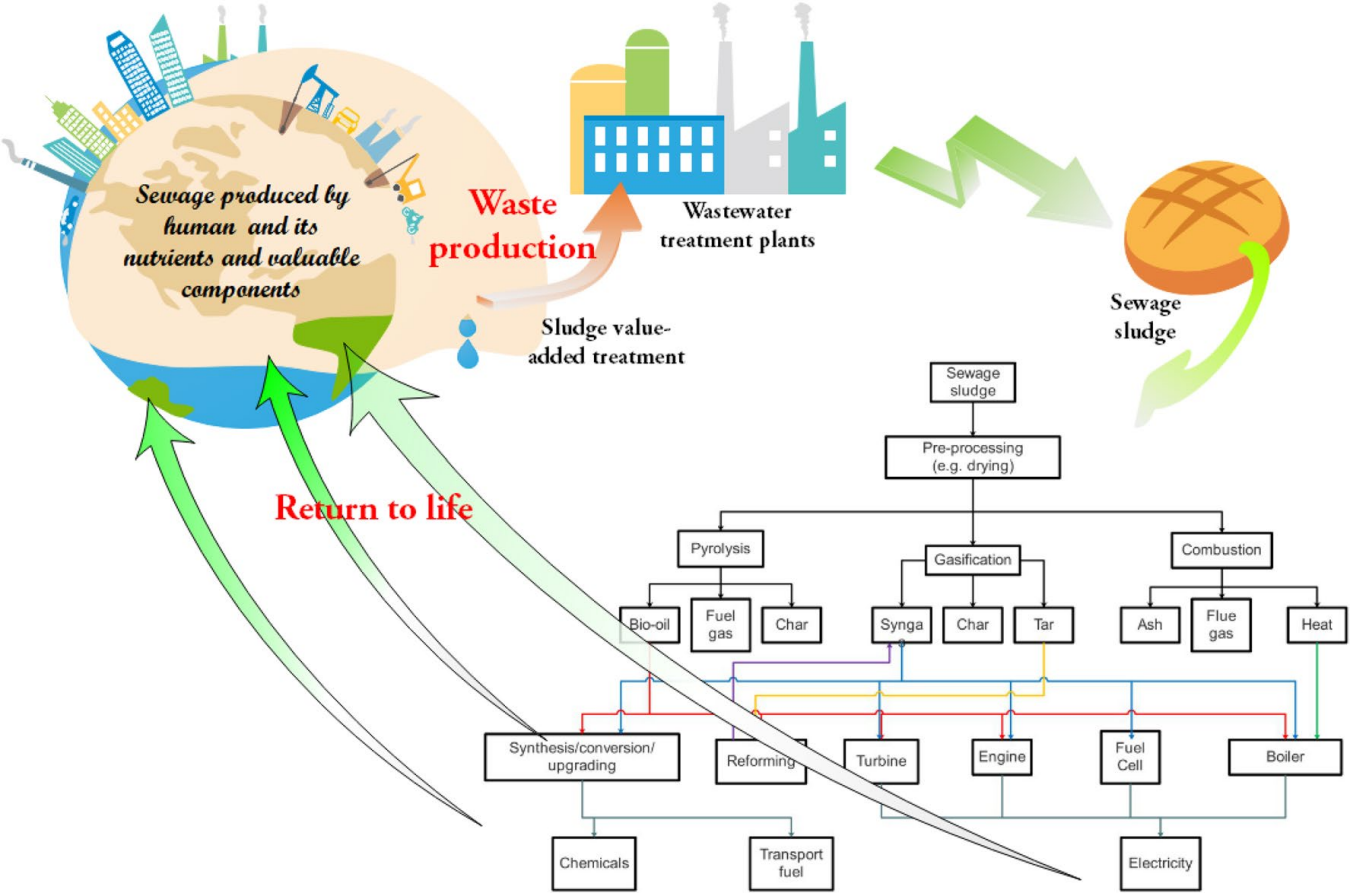
Role of sewage sludge in the water cycle of material circulation in human life^4^.

In fact, dewatered sewage sludge or sewage process factory is an important transfer station in the material cycle of human life. A new abundant, sustainable, and reproducible source resource of energy and chemical could be provided for human if its upstream and downstream cycle path is penetrated (Fig. 1).

Based on the analysis of Fig. 1, we can see that the resource technology for dewatered dewage sludge is a complicated and multi-scale science problem. It needs consider both of water treatment effect in the upstream and a series of target optimizing foundation in the downstream for the resource of dewatered sewage sludge. multi-scale associated research and tackle key problem is a sort of effective method for the similar complicated science problem to actualize technology and industrialization span development, consulting the successful industrialization history of coal liquefication in China. Scale effect is crucial in complicacy problem and substance diversity research. Difference scale often causes the difference of mutual force and then result in qualitative difference of substance performance and its movement law and principle. Kui Wang figured out that any substance movement in complicacy system must involve in substance structural change, while structural change is a multi-level variation from primary structure to advanced structure. Chemistry of levels above molecule is a part of complicacy system chemistry investigate, because complicacy system research must be multi-level. Research of levels above molecule and the followed research of complicacy system may bring chemistry into a new realm^5^. Siliang Zhang reputed that space–time multi-scale characteristic is a common characteristic of all complicacy phenomenal in process engineering. It is quite difficult to find the relation between different scale in the substance transformation research if some different scale of substance phenomenal is chosen as target due to the difficulty of microcosmic and macroscopical data statistical treatment. However, It is probability to acquire essential knowing from appearance and process research or control will be qualitatively changed if such difficults are conquered^6^.

Multi-scale problem acted an important role in the development of modern science. nowadays, it gains comprehensive attention and application in the energy and resource research of biomass. It comes down to different scale of heat transfer^7,8^, compound material and analysis of biomass construction at different scale^9–16^, different scale of thermodynamics and dynamics for biomass treatment process^6,15–25^, different scale of chemistry mechanism^26–32^ and multi-scale coupling in biomass treatment^32–45^. Mushrif figured that multi-scale tool is a effective means in biomass transform technology^46^, Coupling of multi-scale is more important in the research of energy and resource for biomass^47–59^. but research papers with multi-scale coupling tool in the research of resource of dewatered sewage sludge is still rare. He and Zhang studied the multi-scale construction analysis of hydrothermal coke which was maken from dewatered dewage sludge^60,61^, and which is pioneering in the research of resource of dewatered dewage sludge with multi-scale analysis tool. balance between forepart water treatment and followed dewatered dewage sludge is used as a resource of organic biomass in the carbon sink of earth is very important because it is a key step. therefore, resource of dewatered sewage sludge is a multi-scale and multi-levels science problem, and application of analysis tools of multi-scale to the whole process maybe promote the research develops to a new level. Musun Guo figured out that cosmic substance construction there are 5 big levels, namely below atom level, molecule and its gather state level, biologic level, telluric level and cosmic level^62^. we showed the referred multi-scale problems in the process of catalytic crack of dewatered sewage sludge into small chemicals since its generation followed Guo's analysis and generalization for multi-scale problem in his book named <multi-scale effect in the process of substance transform> ^62^.

**Figure 2:**
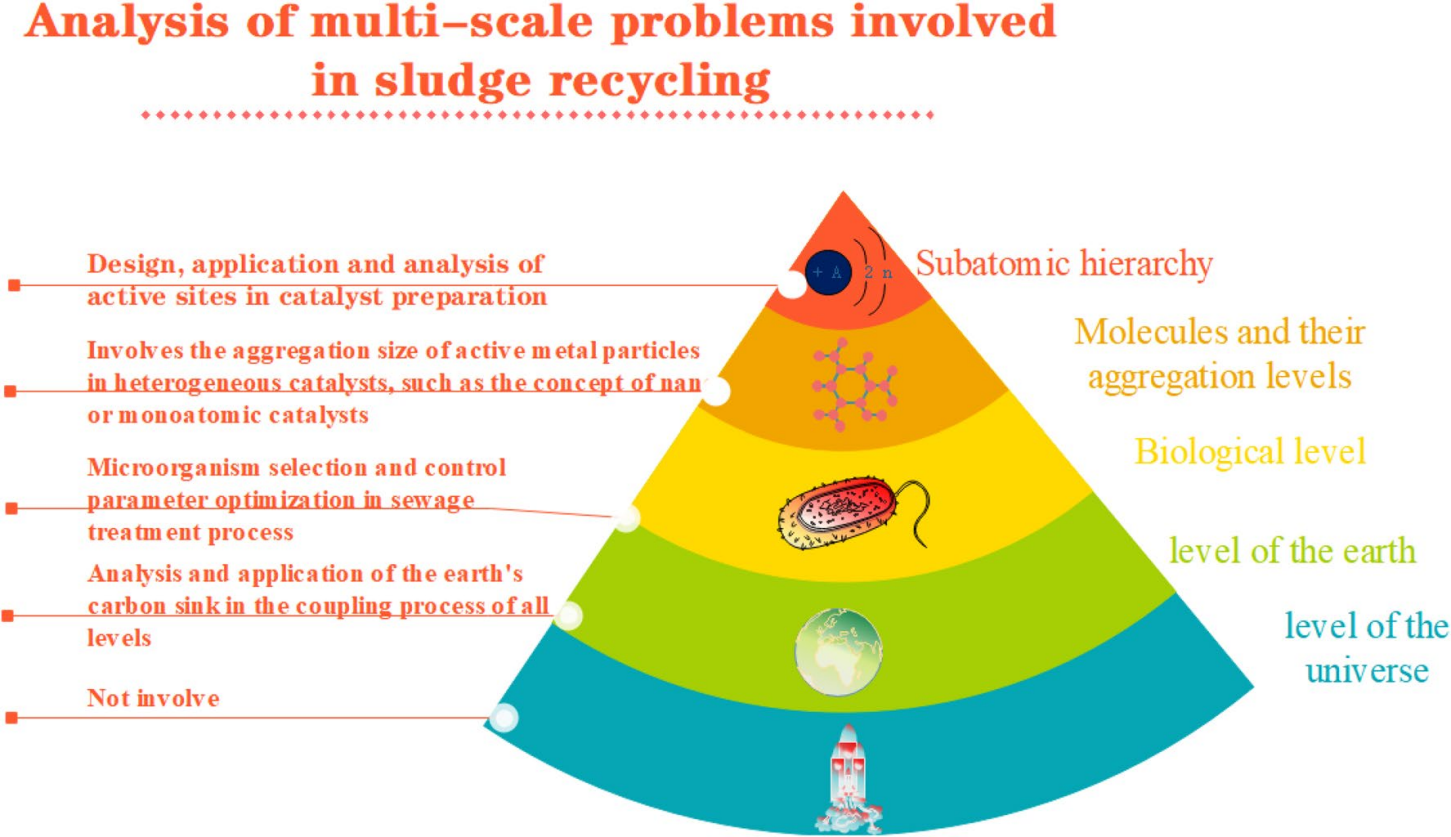
Analysis of multi-scale problems involved in sludge recycling.

Based on this analysis, we found that resource of dewatered dewage sludge is involved in all levels except of the universe level (Fig. 2). research for the coupling and correlation of biologic level and molecule and its gather state level should be the core problem in the complicacy multi-scale problem of the resource of dewatered dewage sludge. Research for this problem not only need to satisfice the technical demand for the forepart water treatment, it also affects the product of the back-end resource of dewatered dewage sludge, for example, the selectivity for certain valuable chemicals. In the technology route of chemical manufacture, thermochemistry treatment of dewatered dewage sludge is a sort of effective technology path, it is a representation of multi-scale problem for resource of dewatered dewage sludge at the level of molecule and its gather state level.

According to the participation degree of oxygen in the chemistry reaction, Wang Yi summarized that there are three technologies, included in pyrolysis, gasification and combustion after advanced process of dewatered dewage sludge such as dryness and so on^63^. Dewatered dewage sludge may produce biomass oil combustible gas and coke if oxygen is isolated. Syngas, coke, and tar could be gasified from dewatered dewage sludge under the atmosphere of part oxygen participation. dewatered dewage sludge may be burned to generate heat and get into the next substance form through exhaust gas and ash if the temperature is enough high and oxygen is excessive. the possible pyrolysis product of dewatered dewage sludge is shown in Table 1 and Tables S3–S9^64^ (in the supporting information) based on literatures and our former researches.

**Table 1:**
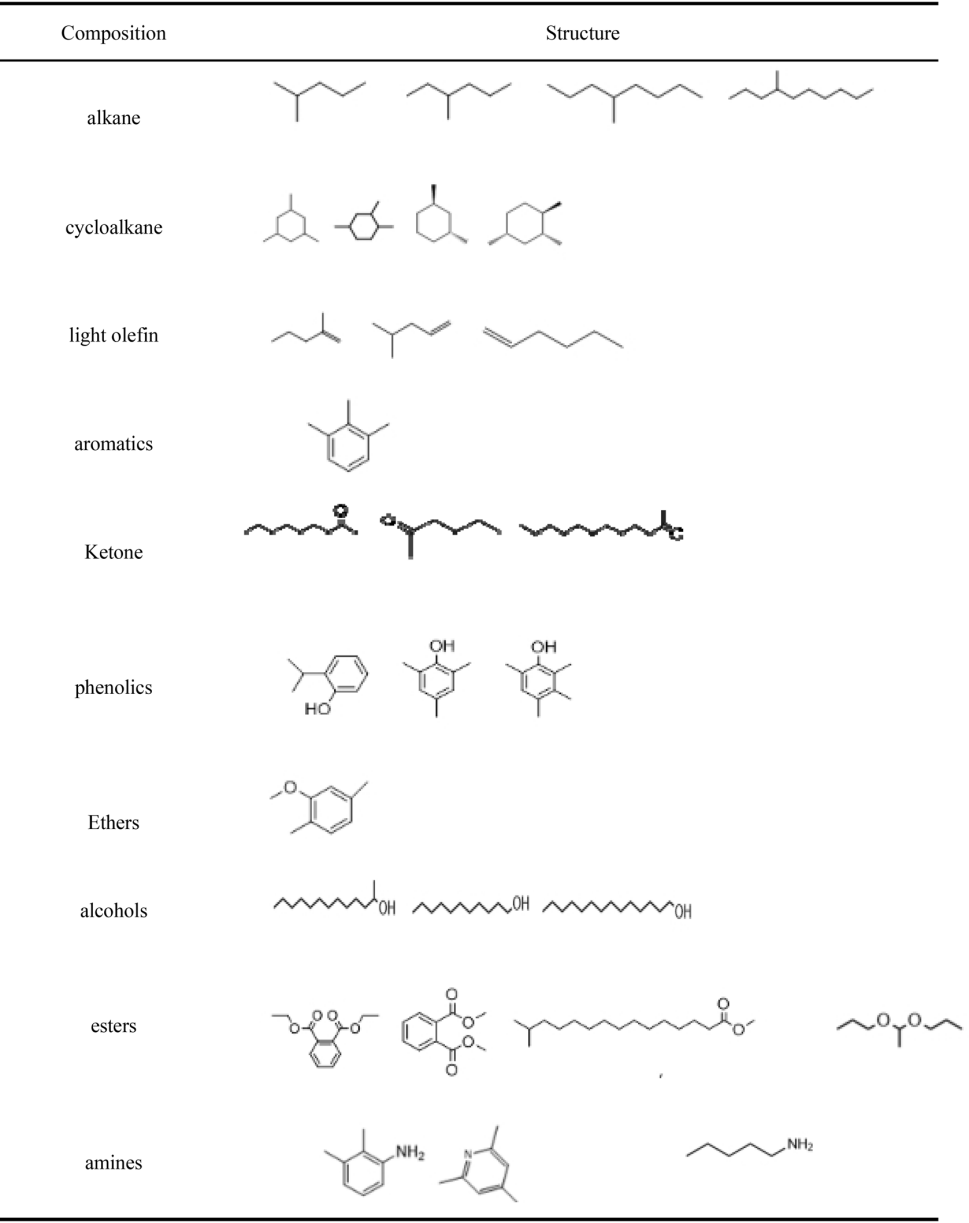
Main components of liquid products of catalytic cracked of sewage sludge.

**Figure 3:**
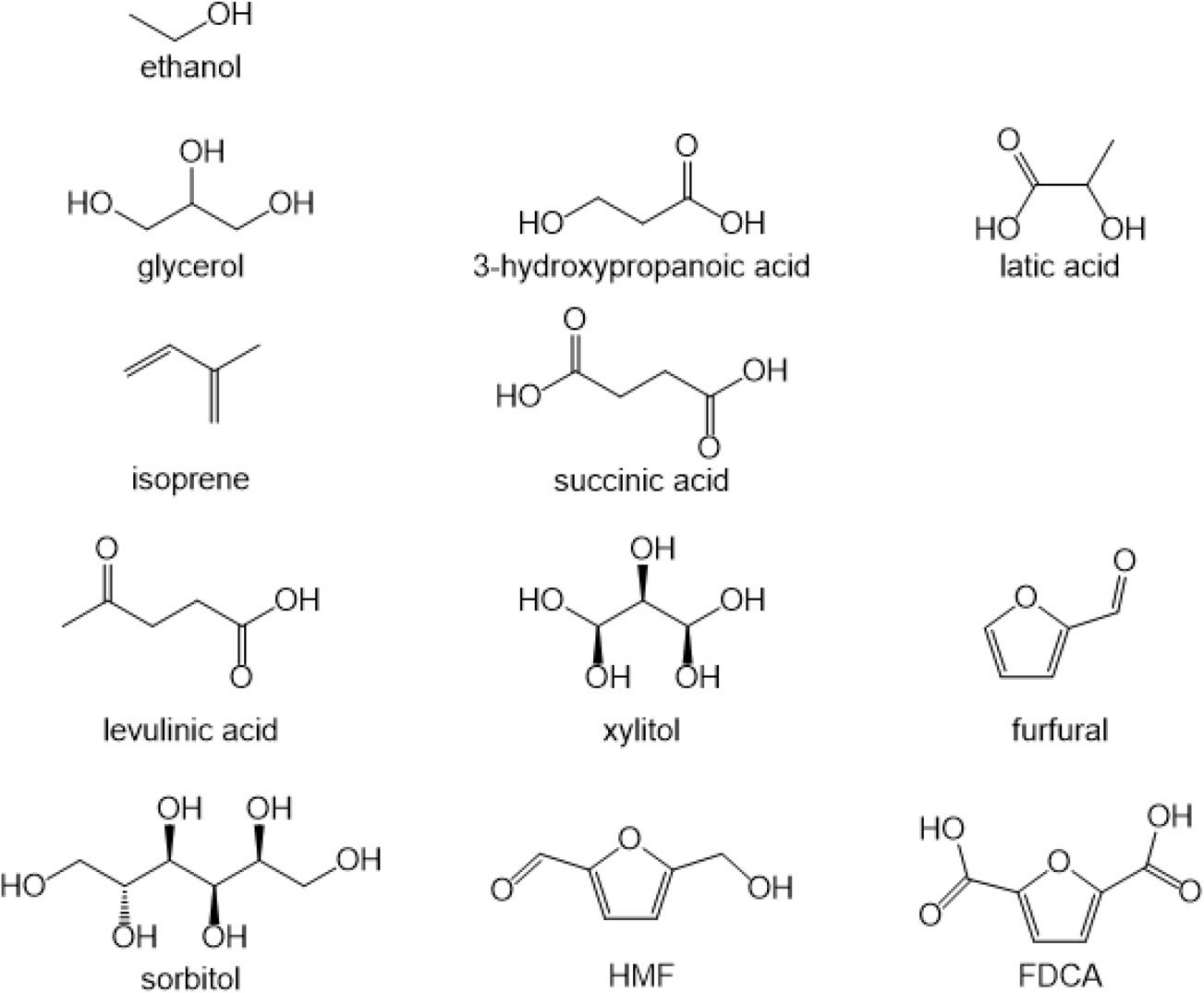
Revised top 12 biobased platform chemicals^25^.

Comparing to the report of 12 key platform chemicals from biomass, which was released by the Pacific Northwest National Key Laboratory and National Renewable Energy Laboratory of America (Fig. 3)^65,66^, we find that the pyrolysis products of dewatered sewage sludge not only includes in essential platform chemicals like glycerol etc. , essential chemical middle product like levoglucosan, and terminal function chemicals like pyridine or 4-acetylaminopyridine also could be produced.

In summary, the downstream research of sludge focuses on the preparation of biochar from sludge and its application^67–72^, energy and nutrient recovery^73–76^, removal of toxic and harmful substances and heavy metals from sludge^77–81^. There are also some reports on the microbial ecological structure of sludge^82–84^, the pyrolysis performance of sludge^85–87^ and its mechanism^88–91^, and even some industrial practices^92^. However, No literatures focusing on the research of production of small molecular chemicals from sewage sludge directly, and the only few papers focused on the use of fermentation means^93–97^. The use of heterogeneous catalysis has not been reported, so this paper might be the first paper to systematic study the possibility of produce small molecular chemicals from sewage sludge.

This paper will report the catalytic cracking and hydrogenation of dewatered sewage sludge by coupling the molecule and its gather state level and biologic level to produce small molecular chemicals, and the effect of sewage sludge source, effect of catalyst and effect of solvent will be discussed carefully from different perspectives.

Three innovations will be done in this paper:

1. Catalytic cracking technology will be applied to the systematic research of dewatered dewage sludge to manufacture high value chemicals, it is hopeful to achieve the target that resource of dewatered dewage sludge to produce high added value chemistry molecule substance, and make the course of generation of dewatered dewage sludge become a purposeful resource regeneration course, and following propelthe technical routes of disposal and treatment of dewatered dewage sludge become greener and more sustainable.
2. In this technology treatment course, overwhelming majority fraction of dewatered dewage sludge will be transferred into organic chemistry substance, and the heavy metal in dewatered dewage sludge will be naturally enriched and it is possible that it participates in the course of catalysis cracking of dewatered dewage sludge and thereby decrease the dosage of exogenous catalyst and decrease the cost of catalyst preparation and reclaim in the treatment course. At the same time, the heavy metal residue could be reclaimed as a heavy metal resource when the enrichment of heavy metal arrives at to a high level. therefore, the heavy metal in dewatered dewage sludge which applied this method is not any more an environmental risk factor, while it becomes to a valuable or even higher value resource what is sustainable and geener.
3. There is no special condition need to be arrived for the moisture of dewatered dewage sludge in this technology route. On the contrary, the moisture in the dewatered dewage sludge could get into the chemistry molecule as a reactant and thereby avoid the high cost for the dewatering and drying of dewatered dewage sludge in the present disposal method, and the entire atomic economy for the reaction also could be improved.

**Figure 4:**
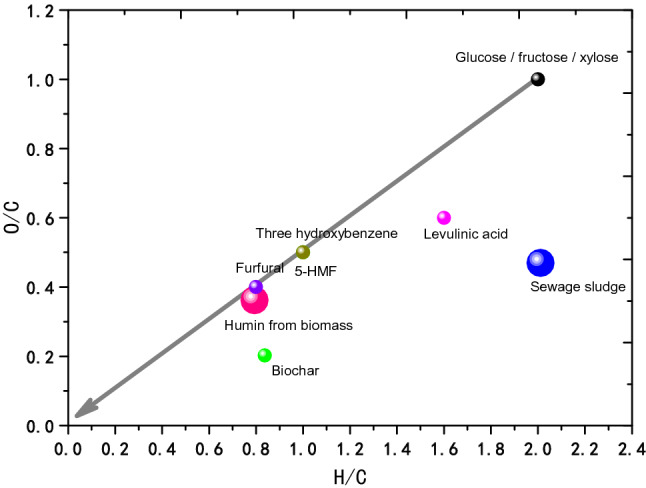
A van Krevelen plot depicting the changes in H/C and O/C ratio during acid-catalysed dehydration of sugars: carbohydrates (black squares), LA (grey squares), HMF and TB (grey triangles), FF (black triangles), water-soluble products and oligomers (black diamonds), humins (grey diamonds), and biochar (black dots). The elemental dehydration reaction is depicted by the grey arrow. The insert shows a magnification of the elemental composition of humins prepared from different feedstocks: gluc = glucose, fruc = fructose, and xyl = xylose.

The most difficult of this research is that the cracking of dewatered dewage sludge relate to a complicated gas–liquid–solid three phase reaction, therefore the commixture and solid to solid mass transfer between dewatered dewage sludge and catalyst maybe become a bottleneck for the enhancement of reactivity and selectivity if heterogeneous catalyst is used. But based on our research on humin (Wang, Y., et al., Catalytic Hydrotreatment of Humins in Mixtures of Formic Acid/2-Propanol with Supported Ruthenium Catalysts. ChemSusChem, 2016. 9(9): pp. 951–961), which is a biomass with similar nature to dewatered dewage sludge (Fig. 4). It is obvious that dewatered dewage sludge has higher O/C and H/C, which enunciate that dewatered dewage sludge has higher catalysis transformation potency than humin. Our research of catalysis crack of humin showed that its transfer rate is up to 77%^98^, so it is possible that the catalysis cracking of dewatered dewage sludge could arrive at higher transfer rate.

In this paper, we report a detailed study on the dewage sludge depolymerisation with heterogeneous catalysts in combination with the effect of the source of dewage sludge, which is a sign of the effect of the microbiology structure, and a hydrogen source (molecular hydrogen, formic acid (FA), isopropanol (IPA)) also was studied in this research. Initial experiments were conducted at standard conditions and the product oils were quantified and characterised in detail using advanced GC techniques and GPC. Subsequently, the effect of microbiology structure was analyzed by 16SrRNA analysis.

## Materials and methods

**Figure 5:**
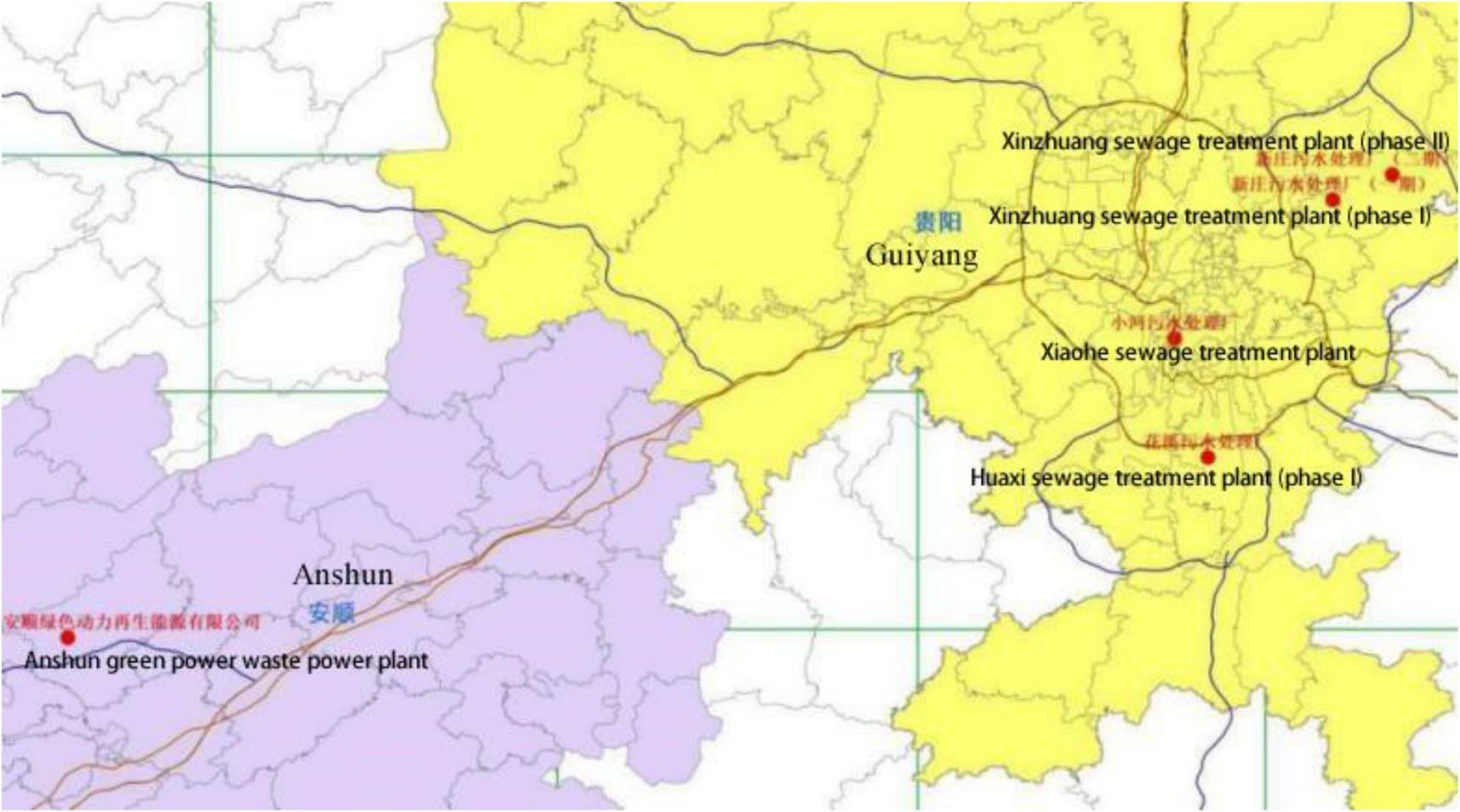
Distribution of sampling points of sewage sludge (Draw with ArcGIS 10.2 for Desktop Version:10.2.0.3348).

Firstly, a standard experiment condition, 30 g IPA, 0.100 g Ru/C, 2.000 g sewage sludge were set up to test the effect of source of sewage sludge, effect of catalyst and effect of solvent. The sewage sludge tested were shown in Fig. 5, which were got from Xinzhuang sewage treatment factory (Phase I and Phase II), Huaxi sewage treatment factory, Xiaohe sewage treatment factory and Anshun green power renewable energy Co., Ltd (Fig. 8), which included all type of dewatered sewage sludge source around Guiyang city.

Here we took Huaxi sewage treatment factory as an example to explain it carefully in the supporting information, and the information of other sample source could be found by contacting the authors.

16SrRNA analysis for the sewage sludge was used to help to appraise the effect of biology, and the samples were listed in Table 2. Catalyst tested were shown in Table 3 and the solvents tested were shown in Table 4.

**Table 2:** 16SrRNA high-throughput analysis samples.

No.	Name of sewage treatment plant	Nature
T-1	Huaxi sewage treatment plant	Aerobic
T-2	Xinzhuang wastewater treatment plant (phase 1)	Aerobic
T-3	Xinzhuang wastewater treatment plant (phase 1)	Anaerobic
T-4	Xiaohe sewage treatment plant	Aerobic
T-5	Anshun green power waste power plant	Aerobic
T-6	Anaerobic biological purification sample	Anaerobic
T-7	Anshun green power waste power plant	Anaerobic

**Table 3:** Catalysts tested in this research.

Name	No.	Manufacturer
Platinum carbon catalyst Water content < 80%	Pt/C 1	Shanghai McLean Biochemical Technology Co., Ltd
Platinum carbon catalyst (Mw)	Pt/C 2	Shanghai McLean Biochemical Technology Co., Ltd
Palladium carbon catalyst	Pd/C	Shanghai McLean Biochemical Technology Co., Ltd
Ruthenium carbon catalyst	Ru/C	Tianjin Hiens Biochemical Technology Co., Ltd
Iridium carbon catalyst	Ir/C	Shanghai McLean Biochemical Technology Co., Ltd
Rhodium carbon catalyst	Rh/C	Shanghai Myrel Chemical Technology Co., Ltd

**Table 4:** Solvents tested in this research.

No.	Solvent
1	Isopropanol
2	Ultra-pure water
3	Isopropanol + formic acid
4	Isopropanol + methanol
5	Isopropanol + ethanol

### Chemicals

All chemicals used in this work were of analytical grade and used without further purification. Some of the chemicals were obtained from the companies listed in Table 3. The catalyst was not pre-activated and used as such.

More information for some chemicals is listed in the supporting information.

### Catalytic pyrolysis of sludge

Firstly the sludge was pretreated with this process: Weigh 200 g of sludge, put it in a 500 mL beaker and seal it with plastic wrap, cut a few holes on the plastic wrap with scissors, place it in a freeze dryer for 48 h, and take it out after vacuum drying. Crush the sludge with a grinder and put it into a beaker and seal it with plastic wrap.

The hydrogenation experiments were performed in a whf-0.1 laboratory magnetic stirring reactor and its parameters were shown in Table S1 (in the supporting information).The whf series laboratory magnetic stirring reactor is a stirring reaction device for chemical reaction of liquid–liquid, gas–liquid, liquid–solid or gas–liquid–solid three-phase chemical materials, which can make chemicals stir well under the given pressure and temperature to enhance the mass and heat transfer.

**Figure 6:**
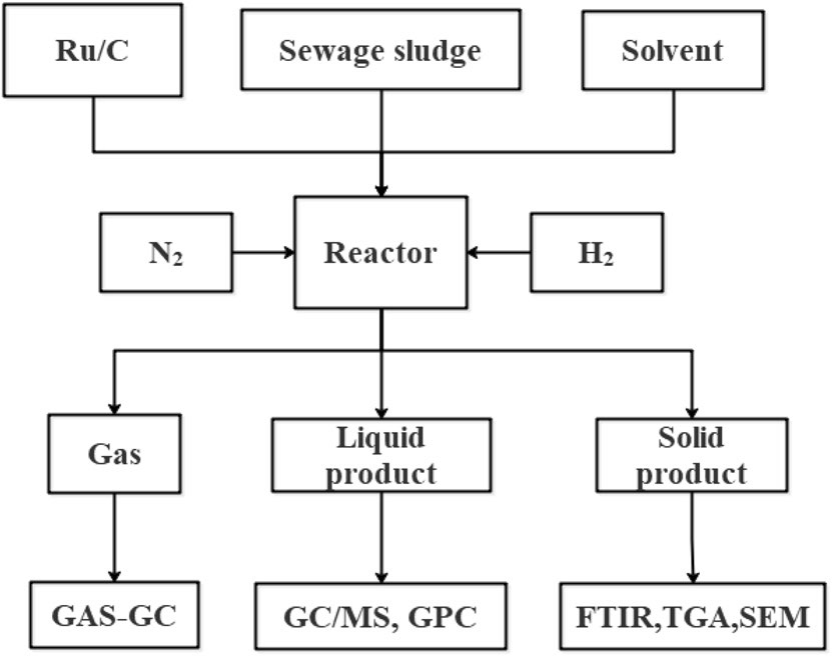
Overview of the experimental procedure for a catalytic liquefaction experiment.

A representative example of an experiment is schematically given in Fig. 6:

- Dehydrated compressed medicated sewage sludge from Huaxi Sewage Treatment Plant which has been freeze-dried beforehand: 2.00 g;
- Catalyst: 0.100 g;
- Solvent: 30.000 g isopropanol for the reseaches of the effect of catalyst and sewage sludge source, and in the reseach of solvents it was set in the ratio of the fellowing data:
- First time: 30.000 g isopropanol;
- Second time: 30.000 g ultrapure water;
- Third time: isopropanol: 9.000 g, formic acid: 9.000 g;
- Fourth time: isopropanol: 9.000 g, methanol: 9.000 g;
- Fifth time: isopropanol: 9.000 g, ethanol: 9.000 g.

The standard operation process is shown in the supporting infromation.

Product collection includes gas phase, liquid phase, and solid phase:

1. Gas phase products: After the reaction is finished, connect a 1L gas bag to the gas release valve, and slowly open the gas collection valve to deflate until the gas bag bulges. After collection, store it for backup and measure the gas volume by drainage method.
2. Liquid phase-solid phase product: when the pressure gauge shows that the pressure is 0, open the reactor, and collect the mixture in the reactor with a well-centrifuge tube; put it into the centrifuge and set the centrifuge speed at 5000 rpm, t = 30 min, and then start centrifuge, collect the supernatant after centrifugation in a 20 ml sample bottle (that is, the liquid phase), put the remaining sludge in an oven at 75 °C for 48 h, and leave the remaining material after drying, which is the solid phase product.

The analytical methods were shown in the sourpporting in formation.

### Definitions

1. Calculation of sludge conversion rate

$$ n = \frac{{m_{1} - \left( {\left. {M - m_{2} - m_{3} } \right)} \right.}}{{m_{1} }} * 100\% $$

where *n*, sludge conversion rate; *M*_,_ total weight of dried sewagle sludge sample, catalyst and centrifuge tube, g; *m*_1_, weight of sludge added in the reaction, g; *m*_2_, centrifuge tube weight, g; *m*_3, catalyst weight, g;_

2. Sludge conversion rate (exclude medicine)

Before the dehydration of sewage sludge, drugs are usually added in the sewage sludge for dehydration pretreatment. So, the pyrolyzed sludge which were collected directly from the plants usually contain some drugs, which will affect the conversion rate of catalytic hydropyrolysis of sewage sludge.

Because Huaxi Sewage Treatment Plant, Xiaohe Sewage Treatment Plant and Anshun Green Powered Waste Incineration Power Plant used pam (polyacrylamide) as the agent, the dosage ratio was 1/1000, so the effect of the agent on the results was negligible. However, the chemical used in Xinzhuang Sewage Treatment Plant is lime, and the dosage is 0.2, and the possible forms of lime after adding sludge are calcium hydroxide, calcium carbonate, and calcium oxide. Since calcium carbonate is cracked into calcium oxide and carbon dioxide at 800–900 °C, the pyrolysis temperature of calcium oxide is higher than 900 °C, while the maximum pyrolysis temperature in these experiments is 360 °C. Therefore, the quality of the dehydrating agent remains the same before and after the experiments. So the dosage of the added agent he Xinzhuang Sewage Treatment Plant has a large effect on the conversion rate. So we recalculate the conversion rate.

Calculation was performed like this:

$$ n = \frac{{m_{1} * (1 - 0.2) - \left( {\left. {M - m_{2} - m_{3} } \right)} \right.}}{{m_{1} * (1 - 0.2)}} * 100\% $$

where *n*, sludge conversion rate; *M*_,_ total weight of dried sewage sludge sample, catalyst and centrifuge tube, g; *m*_1_, weight of sludge added in the reaction, g; *m*_2_, centrifuge tube weight, g; *m*_3_, catalyst weight, g; 0.2, lime dosing ratio in sewage sludge.

3. Sludge conversion rate (after deducting the moisture in the sludge and the effect of adding chemicals)

Because there is some free water and cell-bound water in the sludge, the conversion rate of the sludge pyrolysis should be deducted from the effect of water. The conversion rate of the sludge after deducting the addition of chemicals and water is shown as follows:

$$ \gamma = (1 - \frac{{M - m_{1} - m_{2} }}{m * (1 - \lambda ) * (1 - \kappa )}) * 100\% $$

where γ, Sludge conversion rate; *M*, sludge pyrolysis drying solid phase, catalyst and centrifuge tube total, g; *m*, the mass of sludge added to the reaction, g; *m*_1_, centrifuge tube weight, g; *m*_2_, catalyst weight, g; *λ*, sludge moisture content which is derived from the TGA data; $\kappa$, the input ratio of sludge dehydration agent, Xinzhuang Sewage Treatment Plant Phase I and Phase II are $C_{a} O$, 200 kg/t, that is, the dosing ratio is 0.2. The remaining three sewage treatment plants are dosed with PAM (polyacrylamide), and the dosing ratio is 1/1000, which can be ignored the dosage of the agent.

## Results and discussion

### Sewage sludge characterization

#### Microbial diversity of sewage sludge

rRNA is highly conserved in nucleotide evolution, advanced structure, base composition, and biological functions in the biological evolution process, so it is called bacterial "fossil". This conservation has made rRNA into the current classification of environmental microorganisms. The most useful and commonly used "Molecular Chronograph".

Prokaryotic microorganisms contain three types of rRNA, 23SrRNA, 16SrRNA, and 5SrRNA, among which 16SrRNA is a small subunit ribosomal RNA with a moderate molecular weight and is an ideal research object. The basic principle of 16SrRNA sequence analysis technology is to extract 16SrRNA from environmental microorganism samples The 16S rRNA sequence information is obtained by cloning, sequencing or enzyme digestion, and probe hybridization, and then compared with the sequence data or other data in the 16S rRNA database to identify the types of microorganisms that may be present in the sample.

The chart data for this analysis comes from Shanghai Meiji Cloud Platform (http://www.i-sanger.com).

1. Bacterial analysis

The bacterial and archaea diversity index of urban sludge is shown in Table 5.

**Table 5:** Bacterial diversity index table.

Sample name	OTU	ACE	Chaos	Shannon	Simpson	Coverage
Anshun green powered waste incineration power plant (anaerobic)	227	238.72	238.55	3.34	0.07385	0.99931
Anshun green powered waste incineration power plant (aerobic)	13	19.27	13.50	1.15	0.37068	0.99994
Huaxi sewage treatment plant (aerobic)	1724	1951.19	1972.01	6.16	0.00579	0.98869
Xinzhuang wastewater treatment plant (phase i, anaerobic sample)	1929	2143.93	2162.53	5.94	0.00753	0.99354
Xinzhuang wastewater treatment plant (phase I, aerobic sample)	1911	2111.99	2109.60	6.07	0.00608	0.99289
Xiaohe sewage treatment plant (aerobic sample)	1591	1810.65	1833.63	5.94	0.00704	0.98928

**Figure 7:**
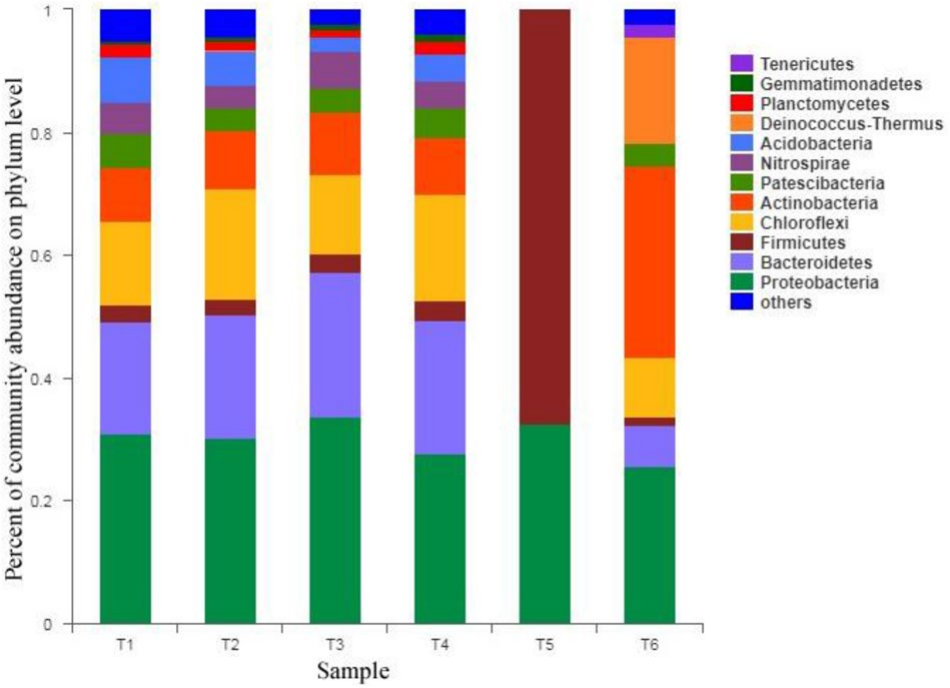
Proportion of bacterial phylum classification abundance/phylum abundance of bacterial.

The bacterial community abundance is shown in Fig. 7:

2. Archaeal analysis

Archaea diversity indicators of sewage sludge are shown in Table 6:

**Table 6:** Archaea diversity index table.

Sample name	OTU	ACE	Chaos	Shannon	Simpson	Coverage
Anshun green powered waste incineration power plant (anaerobic)	61	68.99	66.63	1.25	0.44	0.99971
Anshun green power waste incineration power plant (aerobic sample)	–	–	–	–	–	–
Huaxi wastewater treatment plant (aerobic sample)	125	144.85	142.25	2.19	0.25	0.999271
Xinzhuang wastewater treatment plant (phase I, anaerobic sample)	233	248.26	240.88	2.56	0.23	0.998756
Xinzhuang wastewater treatment plant (phase I, aerobic sample)	153	164.33	160.43	2.96	0.10	0.99925
Xiaohe sewage treatment plant (aerobic sample)	199	225.58	217.50	1.74	0.43	0.998922
Anaerobic purification of samples	49	58.04	58.00	0.65	0.76	0.999601

**Figure 8:**
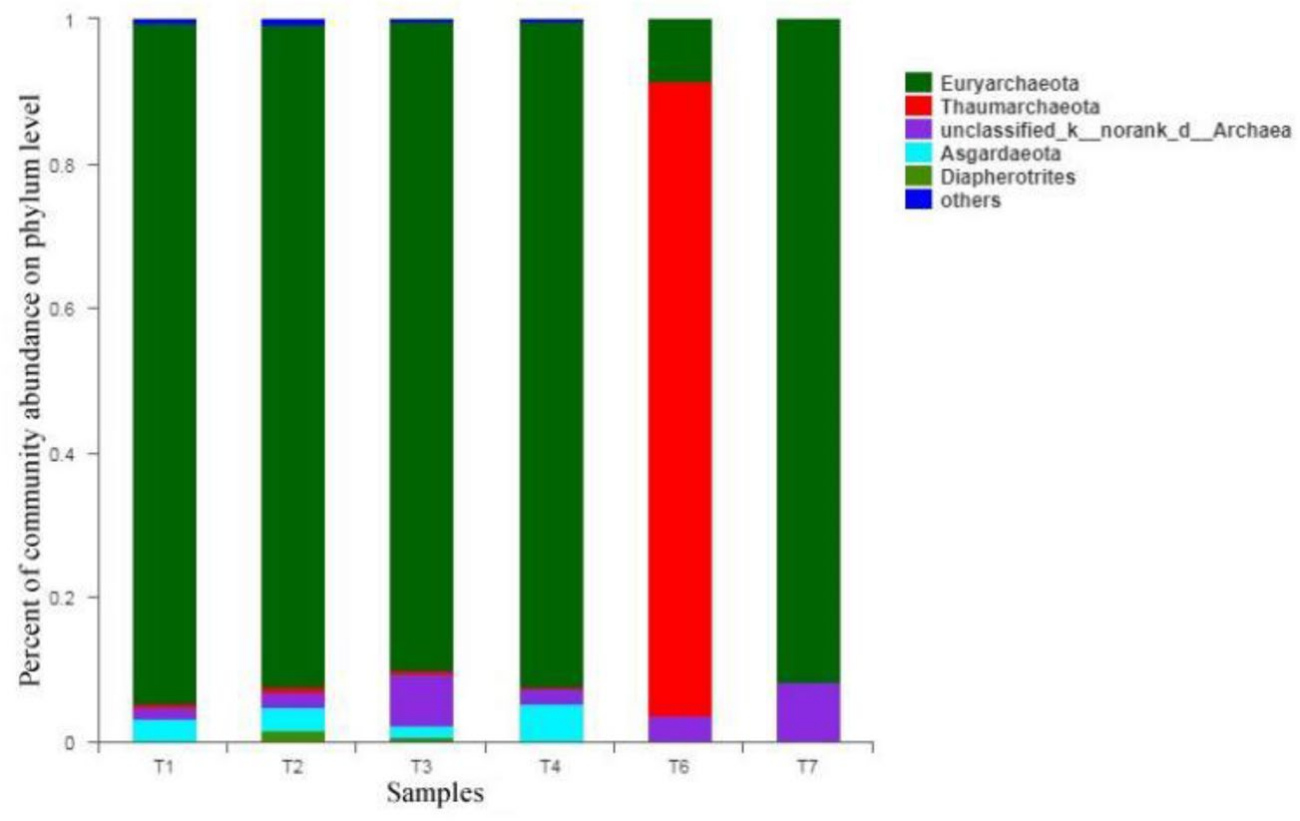
Proportion of Archaea taxonomy abundance.

Archaea community abundance is shown in Fig. 8:

The ACE and Chaos indexes in the table are the microorganisms in the sludge. For the abundance index of the community, the higher the index value is, the higher the richness of the bacteria in the sludge; the Coverage index represents the coverage of each sample library. If the Coverage index is closer to 1, the test result is closer to the actual sample. Results; Shannon and Simpson indices reflect the diversity of microbial communities in the sample.

Real structure; from the perspective of the OTU index, the number of archaea and bacterial microorganisms in several sewage treatment plants are sorted in descending order: Xinzhuang sewage treatment plant, Xiaohe sewage treatment plant, Huaxi sewage treatment plant, Anshun green power waste incineration Power plants; judging from the ACE and Chaos indexes, the order of bacteria and archaeal abundance in several sewage treatment plants shows a consistent rule, from high to low: Xinzhuang sewage treatment plant, Xiaohe sewage treatment plant, Huaxi sewage treatment plant, Anshun green power waste incineration power plant; judging from the Shannon index, the archaeal biodiversity of each sewage treatment plant is Xinzhuang Wastewater Treatment Plant, Huaxi Wastewater Treatment Plant, Xiaohe Wastewater Treatment Plant, and Anshun Green Power Waste Incineration Power Plant. The bacterial biodiversity in descending order is: Huaxi Wastewater Treatment Plant, Xinzhuang Wastewater Treatment Plant, Xiaohe Wastewater Treatment Plant, Anshun Green Power Waste Incineration Power Plant; According to the Simpson index, the proportion of the detected bacteria and archaea in the total microorganisms in descending order is: Anshun Green Powered Waste Incineration Power Plant, Xiaohe Sewage Treatment Plant, Huaxi Sewage Treatment Plant, and Xinzhuang Sewage Treatment Plant.

Pyrosequencing results of bacterial and archaeal communities were shown in Table 6. The OTUs for bacteria obtained from different samples were 227,13,1724,1929,1911,1591 respectively. For archaeal, OTUs of the samples were communities 61, –, 125,233,153,199,49, excluding the Ansun sample (aerobic). The observed ACE and Chao showed that almost all phylotypes could be found in the samples. The coverage of both communities was also over 98%, which indicated that the sequencing results are reasonable.

Shannon and Simpson indices were used to estimate the samples’ community diversities. High Shannon index is a signal of more diverse for the microbial community, while Simpson index should be low. Here Shannon and Simpson index were almost the same except for Ansun samples. There were the lowest Shannon index and highest Simpson index in these samples showed that their bacterial diversity was the lowest.

Archaeal diversity of the Ansun sample (anaerobic) and Xiaohe sample (aerobic) was lower than that of other samples. Shannon and Simpson indexes for Ansun sample (anaerobic) and Xiaohe sample (aerobic) were 1.25 with 1.74 and 0.44 with 0.43. Other samples had almost the same value from 2.19 to 2.96(Shannon index) and from 0.10 to 0.23(Simpson index).

#### FTIR characterization

Fourier transform infrared spectroscopy (FTIR) can perform qualitative analysis of substances, and functional groups on the surface of the sewage sludge may adsorb some impurities, so it is necessary to perform FTIR analysis on the sewage sludge.

**Figure 9:**
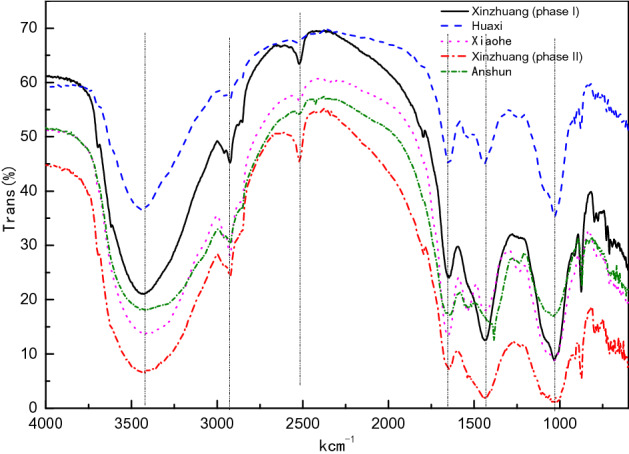
Infrared spectrum of sewage sludge.

Infrared spectra of sewage sludge samples from various sewage treatment plants are shown in Fig. 9:

Based on the OMNIC analysis for the FTIR data of sewage sludge, the functional groups of each sewage sludge from different source are shown in Table 7:

**Table 7:** Summary of functional groups in sewage sludge from different source.

Sewage sludge treatment plant	Functional group
Huaxi sewage treatment plant	Primary aliphatic amides, inorganic phosphates, primary aliphatic alcohols
Xinzhuang wastewater treatment plant (phase 1)	Inorganic carbonate, inorganic phosphates, primary aliphatic alcohols
Xinzhuang wastewater treatment plant (phase II)	Inorganic carbonate, inorganic phosphates, primary aliphatic alcohols
Xiaohe sewage treatment plant	Inorganic phosphates, aliphatic secondary amides, primary aliphatic alcohols
Anshun green power waste power plant	Inorganic nitrites, primary aliphatic amides

**Figure 10:**
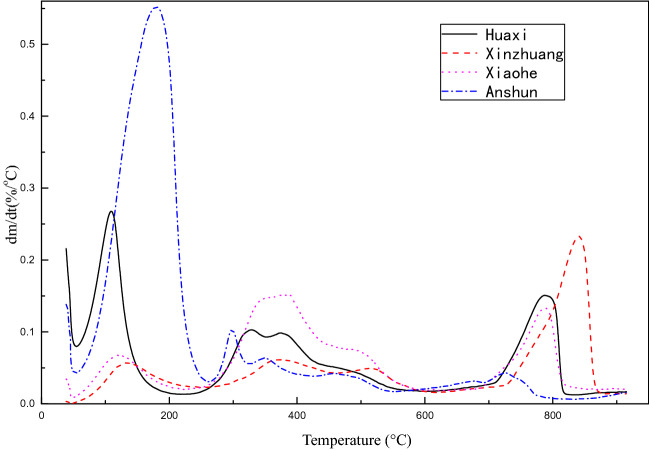
Sludge dtg diagram.

Based on the analysis of Fig. 10, it is concluded that all of the samples from the five sewage treatment plants have a relatively strong absorption peak between 3000 cm^−1^ and 3750 cm^−1^, which is due to the characteristic peak of –NH and –OH stretching vibration; 3000 cm^−1^ is the absorption band that formed by –CH in aromatic hydrocarbons and –CH_3_, –CH_2_ stretching vibration in aliphatic^99^; The absorption band at about 2500 cm^−1^–2650 cm^−1^ is formed due to -SH stretching vibration; the absorption peak at 1600 cm^−1^–1900 cm^−1^ is mainly due to the characteristic peak of C=O stretching vibration ; Peak at 1200 cm^-1^ is the characteristic absorption peak of COC, CO stretching vibration and –OH flexural vibration in alcohol, phenol, and carboxylic acid; the absorption bands at about 1100 cm^−1^ are Si–OC or Si–O–Si stretching vibration peak.From the FTIR diagram of the sludge in Fig. 10, we can see that there is a strong resolution band around 1000 cm^−1^, which may be due to -CO stretching and -OH deformation vibration, at 1500 cm^−1^,the surrounding absorption peak and a band at about 1600 cm^−1^ are formed by CH bending vibration and C=C stretching vibration, so it can be proved that aromatics and phenyl groups exist, and the peak around 1600 cm^−1^ is not only related to N=N, it also related to aliphatic nitro and nitroso compounds^13^At about 3500 cm^−1^, there is a broad absorption peak, which means that there are lots of hydroxyl groups, which indicates that the molecules contains amide components, because the NH tensile vibration at about 3500 cm^−1^ and the 1600 cm^−1^ Carbonyl C=O, and the peaks generated by NH shear and CN tensile vibration around 1400 cm^−1^ can prove the presence of amide components^14^.

#### TGA characterization

**Figure 11:**
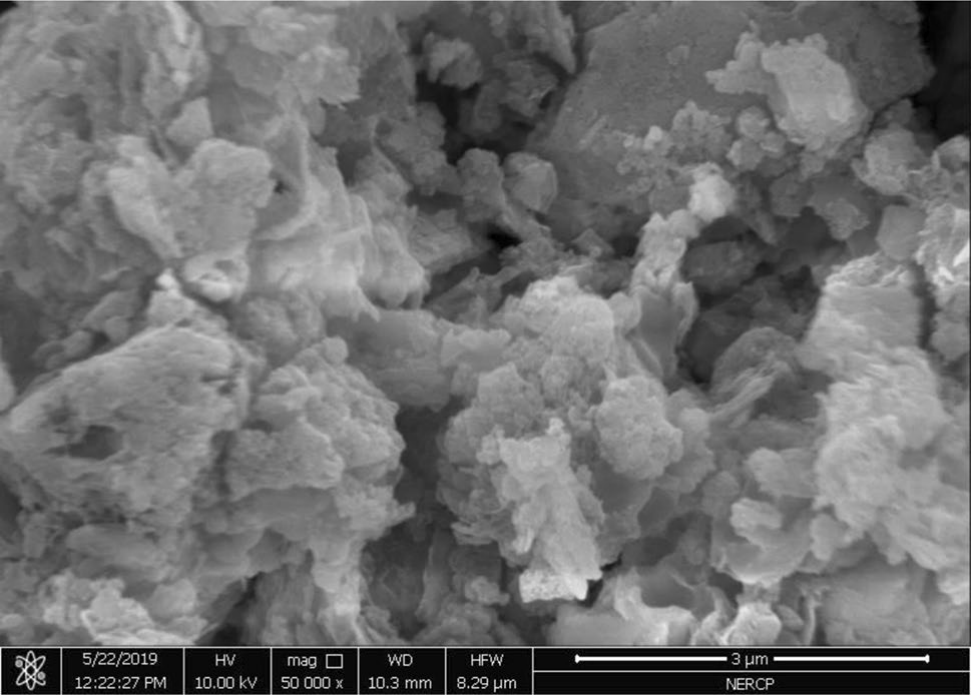
Electron microscope scan of dried sludge.

The thermogravimetric curve of sewage sludge from different source were shown in Fig. 10:

It is known from Fig. 11 that all the samples have a high weight loss rate at 100 °C. Because the gasification temperature of water is 100 °C, it can be determined that the peak that occurs there is caused by water loss; The peaks appearing at 200–500 °C are the pyrolysis of organic matter in sewage sludge, and the peaks at 700–900 °C are macromolecular refractory substances such as cellulose and humus(Table 8)^100^.

**Table 8:** Sludge combustion weightless substances-temperature correspondence table.

Temperature/°C	50–150	150–530	530–670	700–900
Decompose matter	Water	Volatile matter	Fixed carbon	Refractory organic matter

The thermogravimetric peak trend of all samples is roughly same, but there are also some differences:

1. The sludge from Xinzhuang Sewage Treatment Plant showed a small peak of weight loss before 700 °C. This is because Xinzhuang Sewage Treatment Plant added lime with a dosing ratio of 0.2 during the sludge treatment. A large amount of heat makes the water and some organic matter in the sludge less decomposed, and the proportion of non-degradable organic matter in the sludge increases, so the peak of weight loss rate of other sewage treatment plants appears around 800 °C;
2. The samples from Huaxi Sewage Treatment Plant and the Anshun Green Powered Waste Incineration Power Plant showed a more pronounced peak at about 100 °C, which indicates that the decemented samples of the Huaxi Sewage Treatment Plant and the Anshun Green Powered Waste Incineration Power Plant still contain a relatively large proportion of moisture.
3. Sample from Huaxi Sewage Treatment Plant sludge has a smaller weightless peak at 700–900 °C than other sewage treatment plants, which may be related to the treatment process (sbr) of Huaxi Sewage Treatment Plant, compared with others which is smaller at 700–900 °C, The weightless peak is the Xiaohe Sewage Treatment Plant.The wastewater treatment process is also SBR. It can be concluded that the SBR process contains more microorganisms that can degrade difficult-to-degrade organic substances.

As shown in Fig. 11, when the temperature is in the range of 50–210 °C, the original sludge loses weight due to the evaporation of moisture; when the temperature is in the range of 300–500 °C, the original and external sludge samples are due to fibers and some polymer compounds, etc. Difficult volatile organic compounds are decomposed to show weight loss^101^; When the temperature is around 800 °C, the weight loss of sludge is because the non-biodegradable humus and cell wall fibers are decomposed^102^.

#### SEM characterization

**Figure 12:**
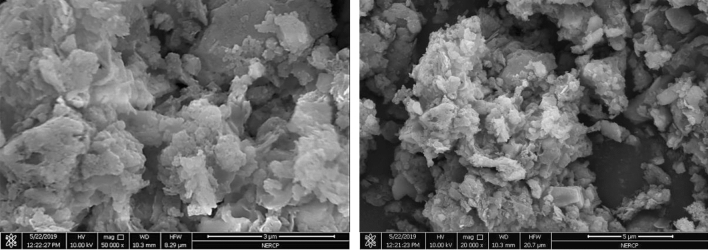
Sem diagram of sludge.

**Figure 13:**
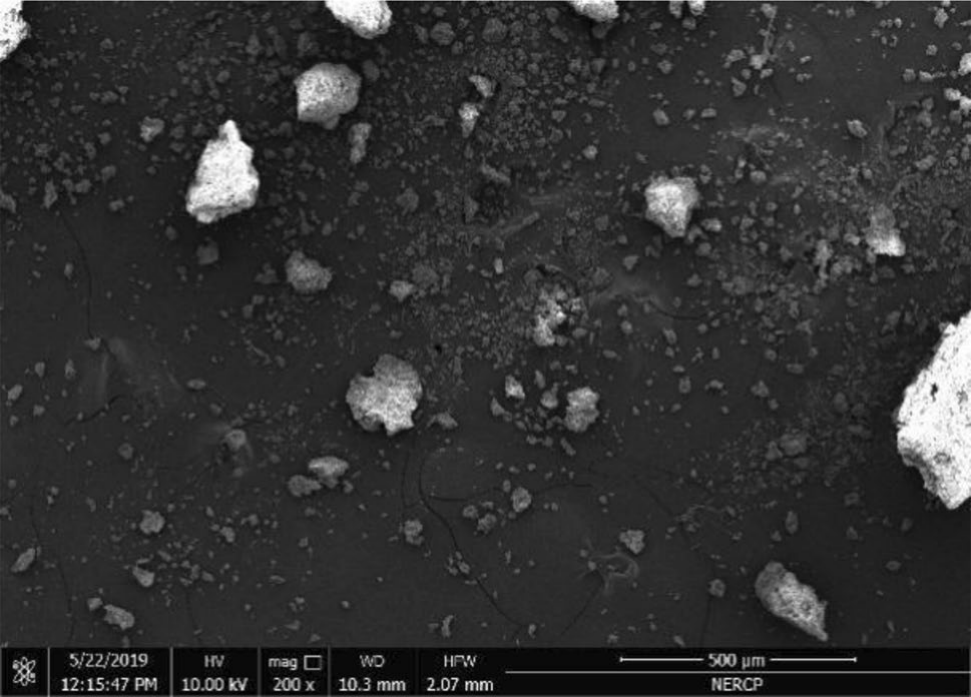
Sem diagram of sludge.

It can be seen from Figs. 11, 12 and 13 that the dried sludge has the characteristics of lamellar and pore-like characteristics. These characteristics indicate that the specific surface area of the sludge is large and the adsorption performance is good. It can be adsorbed more during the sewage treatment process. More bacteria and organic matter, increasing the treatment effect of the sewage treatment plant.

**Figure 14:**
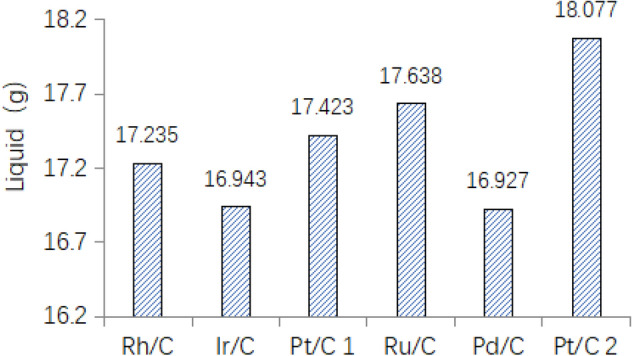
Mass change of liquid product under different catalyst conditions.

Figure 14 is a scanning electron microscope image of the original sludge. It can be seen from the figure that the surface of the mud sample is uneven, the particles are dense and stick together in a mass, and there is basically no microporous structure.

From the Figs. 13 and 14 sludge SEM images, the microstructure of the sludge is nodular and is a large polymer, like foam, with an uneven shape and surface.

### Conversion rate of sewage sludge

Conversion rate of sewage sludge from the five sewage treatment plants was shown in Table 9:

**Table 9:** Urban sludge pyrolysis conversion rate.

Huaxi sewage treatment plant (%)	Xinzhuang wastewater treatment plant (phase 1) (%)	Xinzhuang wastewater treatment plant (phase II) (%)	Xiaohe sewage treatment plant (%)	Anshun green power waste power plant (%)
58.27	56.11	42.41	62.71	83.72

Sewage sludge conversion rate (except medicine) was shown in Table 10:

**Table 10:** Sewagle sludge conversion rate after deducting the effect of added chemicals.

Huaxi sewage treatment plant (%)	Xinzhuang wastewater treatment plant (phase 1) (%)	Xinzhuang wastewater treatment plant (phase II) (%)	Xiaohe sewage treatment plant (%)	Anshun green power waste power plant (%)
58.27	51.74	38.16	62.71	83.72

Conversion rate of sewage sludge after exclude the effect of water is shown in Table 11:

**Table 11:** Conversion rate of sewage sludge with water removed.

Factory name	Huaxi sewage treatment plant (%)	Xinzhuang wastewater treatment plant (phase 1) (%)	Xinzhuang wastewater treatment plant (Phase II) (%)	Xiaohe sewage treatment plant (%)	Anshun green power waste power plant (%)
Moisture content	16.448	5.329	5.329	6.563	55.329
Conversion rate	44.81	49.03	34.68	55.29	53.51

Based on the data of Tables 9, 10 and 11, we can see that after the pyrolysis and hydrogenation of sewage sludge, all sewage sludges are cracked into other substances, and the highest conversion rate is 83.72%, which was the sample from Anshun Green Powered Waste Power Plant. It can be known from Table 11 that after deducting the effect of the chemicals in the sludge, the sludge from Xinzhuang Sewage Treatment Plant has a smaller conversion rate than Table 10. It can be known from Table 10 that after deducting the effects of chemicals and water in municipal sludge, all the sludge conversion rate showed downward trend.

Effect of catalyst to conversion rate:

Firstly, the trend of liquid phase quality under different catalysts was shown in Fig. 14. the weight of liquid phase in the reactor is different, but they were almost between 16 g ~ 19 g, which is at the same level.

**Figure 15:**
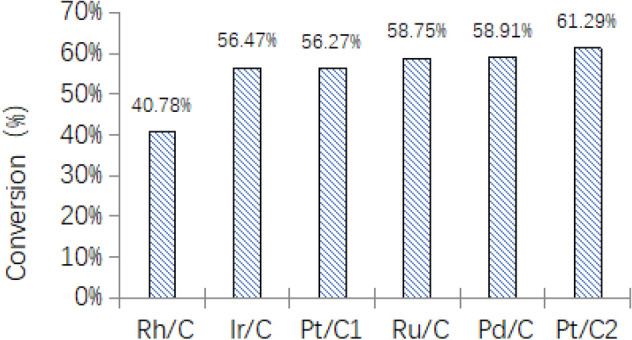
Apparent conversion of sludge.

The trend of sludge conversion rate under different catalyst condition was shown in Fig. 15.

It is known that after the rhodium-carbon catalyst catalyzes sludge, the conversion efficiency of the sludge is only 40.78%, and the conversion rate of others is about 56% ~ 62%.

**Figure 16:**
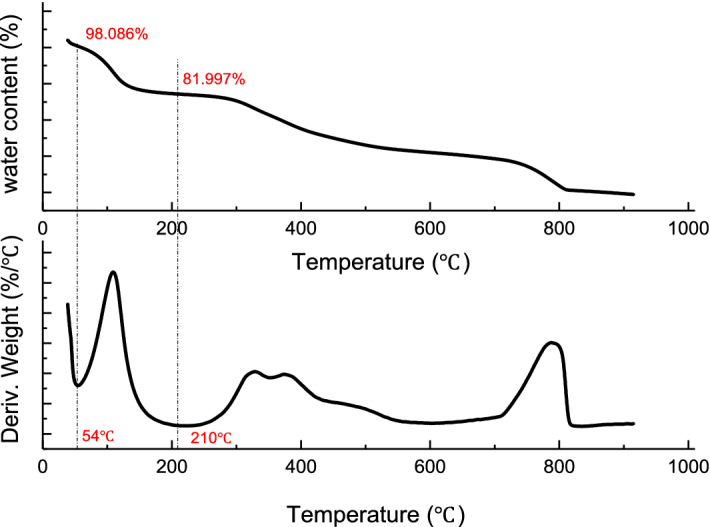
Water content of raw sludge.

Based on the tga data of Huaxi Sewage Treatment Plant sludge after freeze drying is shown in Fig. 16.

**Figure 17:**
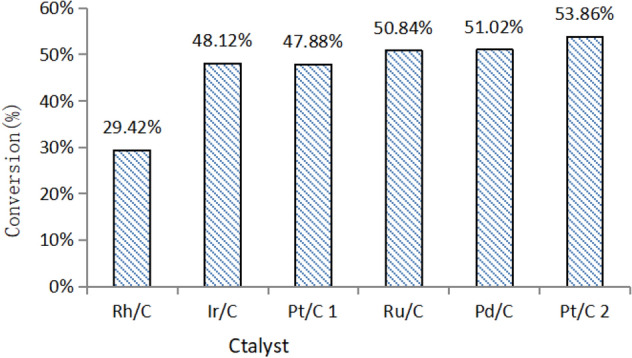
Theoretical dry matter conversion rate of sludge.

It is known that the moisture content of the original sludge is 98.086%-81.997% = 16.089%. So, we could calculate the theoretical dry matter conversion rate of sewage sludge (Fig. 17), and the actual sludge amount and sludge conversion rate in the experiments are calculated as follows:

$$ Drysludge\,mass = Original\,sludge\,mass\, \times \,(1 - moisture\,content(\%)) $$

$$ Conversion\,rate = \frac{{Dry\,sludge - Residual\,sludge}}{{Dry\,sludge}} \times 100\% $$

The results are shown in Table 12 and Fig. 17. Based on the analysis of Table 12 and Fig. 17, we can know that except for the rhodium-carbon catalyt, which only has a conversion rate as low as 29.42%. The others catalysts had a higher conversion rate from 47 to 54%, the catalytic efficiency is almost at the same level, and the catalytic effect is relatively high, indicating that different catalysts have a certain effect on the hydropyrolysis of the sewage sludge.

**Table 12:** Sludge conversion rate.

Use catalyst	Original sludge (g)	Moisture content (%)	Dry sludge (g)	Residual sludge (g)	Conversion rate (%)
Rh/C	2.013	16.10	1.689	1.192	29.42
Ir/C	2.017	16.10	1.692	0.878	48.12
Pt/C 1	2.001	16.10	1.679	0.875	47.88
Ru/C	2.056	16.10	1.725	0.848	50.84
Pd/C	2.015	16.10	1.691	0.828	51.02
Pt/C 2	2.064	16.10	1.732	0.799	53.86

Effect of solvents to conversion rate:

Effect of solvents to conversion rate were evaluated using the sewage sludge from Huaxi Sewage Treatment Plant, and the conversion rate of sewage sludge were calculated by the formula as follows:

$$n = \frac{{M - \left( {m1 - m2 - m3} \right)}}{M}{*}100{\text{\% }}$$

n, conversion rate; m1, total mass after drying g; m2, centrifuge tube mass g; m3, catalyst mass g; M, dry sewage sludge mass g.

The results are shown in Table 13.

**Table 13:** Conversion rate of sewage sludge.

Solvent	Dry sewage sludge mass (g)	Centrifuge tube mass (g)	Catalyst (g)	Centrifuge tube after drying + sample mass (g)	Sludge after drying mass(g)	Conversion rate (%)
Isopropanol	2.056	12.827	0.100	13.785	0.858	58
Ultra-pure water	2.003	12.960	0.100	14.915	1.855	7
Isopropanol + formic acid	2.076	12.915	0.100	14.510	1.495	28
Isopropanol + methanol	2.009	12.945	0.104	13.235	0.186	91
Isopropanol + absolute ethanol	2.003	12.900	0.101	13.935	0.934	53

It can be known from Table 13 that the highest conversion rate of sludge is the reaction of using methanol and isopropanol as solvent, and it as high as 91%, while the lowest is the reaction of using ultrapure water as solvent, and it is only 7%. The reason maybe is because some organic compounds in the sludge are insoluble in water, but soluble in organic solvents.

### Characterization of liquid products

#### GPC characterization

**Figure 18:**
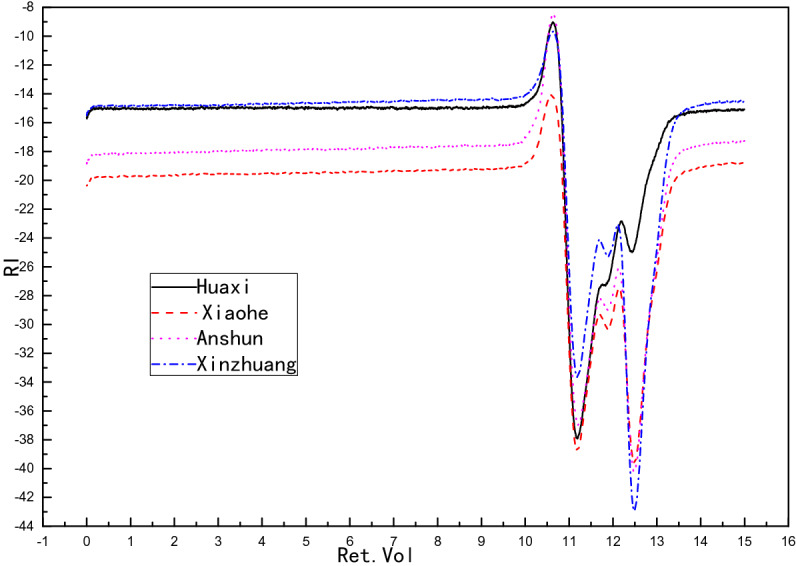
GPC diagram of sludge pyrolysis liquid phase.

Effect of sewage sludge source on the molecular weight:

The results were show in Table 14 and Fig. 18, here we only have two samples of different sewage sludge source had the data because some special experimental reason.

**Table 14:** Sludge liquid product index.

No	M_n_	M_w_	M_z_	M_w_/M_n_
Huaxi sewage treatment plant	1,579,261	2,449,992	4,793,158	1.55
Xinzhuang wastewater treatment plant (phase II)	–	–	–	–
Xiaohe sewage treatment plant	696,288	1,140,625	2,460,355	1.64
Anshun green power waste power plant	–	–	–	–

Based on the sludge liquid product correlation index table (Table 14), the polymer dispersion coefficient M_w_/M_n_ of Huaxi Sewage Treatment Plant and Xiaohe Sewage Treatment Plant are both greater than 1, indicating that the sludge liquid product is a mixture. The average molecular weight of Xiaohe Sewage Treatment Plant is smaller than that of Huaxi Sewage Treatment Plant and the polymer dispersion coefficient is greater than that.

**Figure 19:**
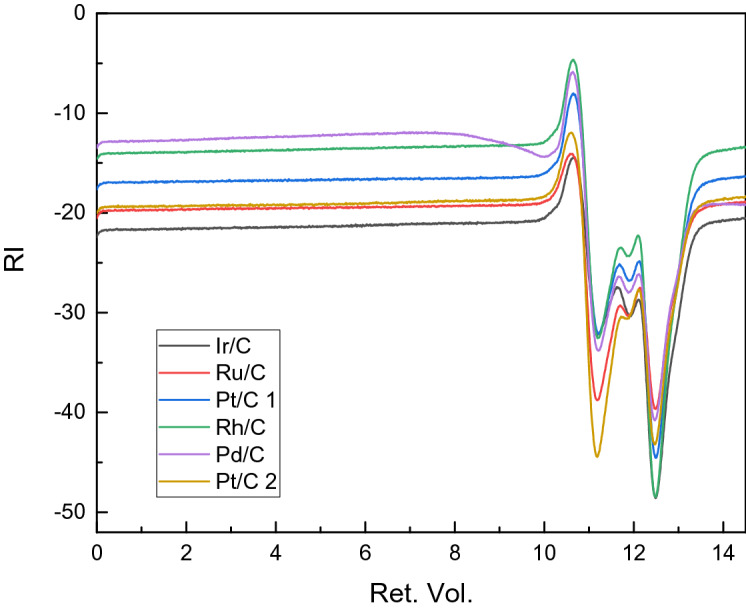
gpc spectra of liquid products under different catalyst conditions.

It can be seen from Fig. 19 that the earlier the peaks, the larger the retention volume. The gpc diagrams of the four sewage treatment plants show similar trends, indicating that the substances contained in each sewage treatment plant are similar.

Effect of catalyst on the molecular weight:

Firstly the mass of liquid phase were calculated as follows: $Liquid{ }massg = Mixture{ }\left( g \right) - dry{ }sludge{ }\left( g \right)$.

After the reaction finished, the liquid phase quality was shown in Table 15.

**Table 15:** Liquid product quality.

Catalyst	Mixture (g)	Dry sludge (g)	Liquid phase mass (g)
Rh/C	18.527	1.292	17.235
Ir/C	17.935	0.992	16.943
Pt/C 1	18.404	0.981	17.423
Ru/C	18.596	0.958	17.638
Pd/C	17.855	0.928	16.927
Pt/C 2	18.976	0.899	18.077

Liquid substance.

Secondly, gpc analysis was performed to analyze the liquid substance to know the information of its molecular weight and substance distribution. The relative data was shown in Fig. 19 and Table 16.

**Table 16:** Molecular weight of liquid product and their pdi.

Catalyst	Mn	Mw	Mz	Mw/Mn
Ir/C	627,288	1,303,814	5,856,110	2.08
Ru/C	1,579,261	2,449,992	4,793,158	1.55
Pt/C 1	353,790	723,524	6,923,026	2.06
Rh/C	423,862	721,300	2,257,133	1.70
Pd/C	673,566	1,367,729	8,113,982	2.03
Pt/C 2	2,569,692	7,441,605	22,278,798	2.90

Based on the analysis of Fig. 19 and Table 16, we could find that all the pdi (pdi:$\frac{{M_{w} }}{{M_{N} }}$. (lymer dispersion index) are greater than 1, indicating that the sample components are unevenly distributed and are composed with mixtures; and the liquid products using Ru/C and Rh/C catalysts have pdi of 1.55 and 1.70 respectively; All of the pdi of the liquid product from Ir/C, Pt/C1, Pd/C catalyst are close to 2.0; while the pdi of the liquid product using Pt/C2 catalyst is as high as 2.90. This shows that the product from Ru/C and Rh/C catalyst has a smaller molecular weight, followed by the liquid product using Ir/C, Pt/C 1, Pd/C catalyst, and the liquid phase product using the Pt/C 2 catalyst has the largest molecular weight.

In gpc, the channel pore size of the substance can be large or small. When the polymer flows through the column, the larger molecules are excluded from the small pore size and can only pass through the large pore size. Short time; small molecules pass through small pores, the rate of passage is slower, and the time to be regretted is longer. Medium-sized molecules are based on large molecules and small molecules. Therefore, in the gpc analysis, if more peaks appear at early part, the molecular weight of the substance is larger. Figure 19 is the retention volume-difference graph, which shows that there is a difference in peak intensity among the samples, but the peak positions are almost same, indicating that there is a selective difference between the catalysts. But the product types are almost same.

**Figure 20:**
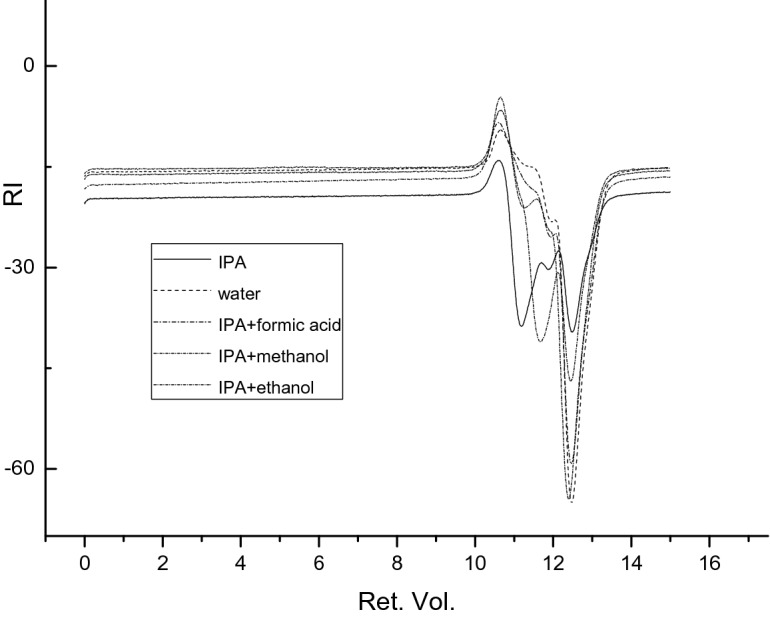
Liquid phase gpc diagram.

Effect of solvent on the molecular weight:

Effect of solvent on the molecular weight were shown in Fig. 20 and the Table 17. The liquid obtained after the catalytic cracking experiments of sludge by adding different solvents showed its position of large peaks appeared at almost same time. Their differences only could be found at a small part area. It means that the types of small molecules obtained after adding different solvents to the catalytic cracking reaction are almost the same.

**Table 17:** GPC experimental data.

Solvent	Mn	Mw	Mz	Mp	PDI
Isopropanol	1,579,261	2,449,992	4,793,158	1,171,446	1.55
Ultra-pure water	–	–	–	–	–
Isopropanol + formic acid	164,611	506,878	6,607,182	171,611	3.08
Isopropanol + methanol	–	–	–	–	–
Isopropanol + absolute ethanol	1,273,263	1,611,583	2,824,465	980,100	1.27

However, PDI represents the dispersion coefficient of the polymer. When the pdi value is greater than 1, it indicates that the peak is wider and the substance is a mixture. The closer the pdi value is to 1, the narrower the peak is. When pdi is equal to 1, it is an absolute monodispersion. It can be known that when the experimental solvent is isopropanol, isopropanol plus formic acid, isopropanol plus anhydrous ethanol, the product in the reaction liquid phase is a mixture, and when ultrapure water and isopropanol plus methanol are used as solvents due to limition of analysis conditions, we did not get valid data. But we can see that the PDI data is changed from 1.27 to 3.08, which means that solvent has a strong effect on the molecular weight of liquid products.

#### GC–MS characterization

**Figure 21:**
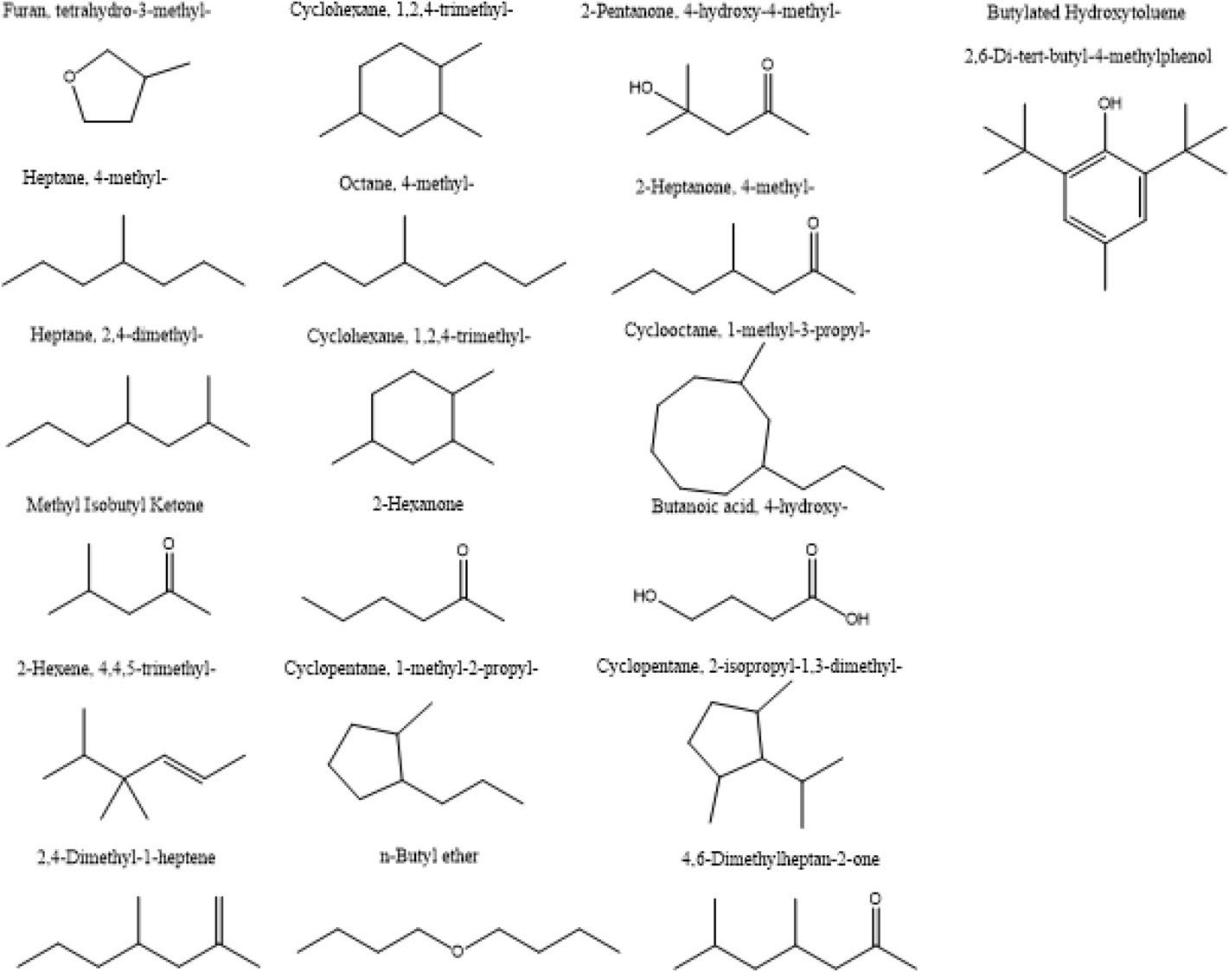
Main substances in the liquid products of sewage sludge catalytic cracked.

The data submitted for inspection are processed by Amdis and compared to Nist2 database to obtain the substances in the liquid products (Fig. 21): The main components of the liquid phase products are alkane, olefin, ketones, aldehydes, phenols, ethers, esters, and some nitrogen-containing compounds (pyridine, aniline) and other organic components. Based on this analysis, we can conclude that it is feasible to produce valuable small molecular substances by catalytic pyrolysis of sewage sludge.

Based on the analysis of the gc–ms spectrum, the liquid products mainly contain the following products (Table 18):

**Table 18:**
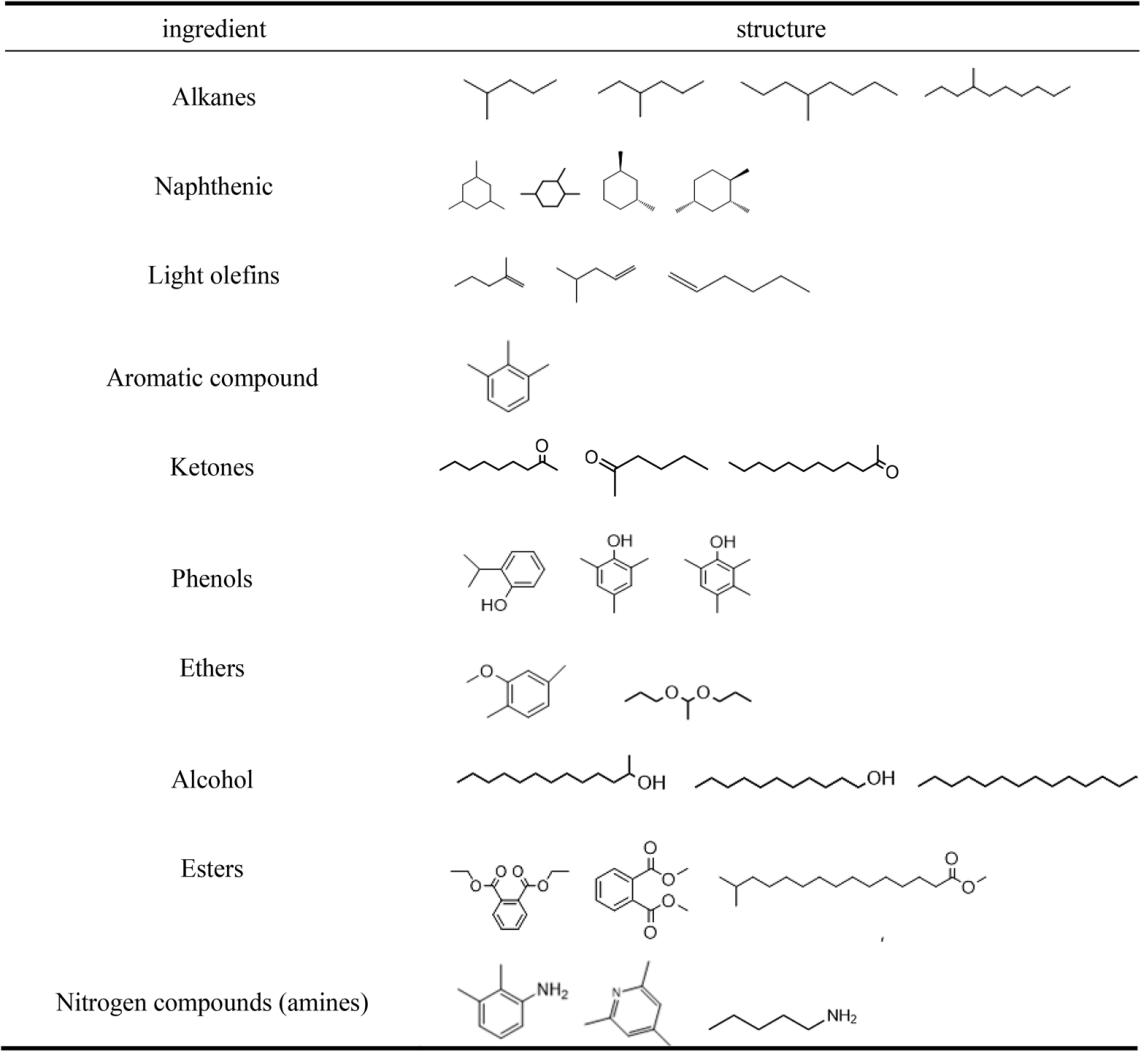
Main components of liquid products.

As a conclusion of these analyses, alkane, olefin, aromatic compound ketones, aldehydes, phenols, ethers, esters, and some nitrogen-containing compounds (pyridine, aniline) and other organic components were the main components of the liquid product. These results also showed that valuable small molecular chemicals could be produced from catalytic pyrolysis of sewage sludge.

**Figure 22:**
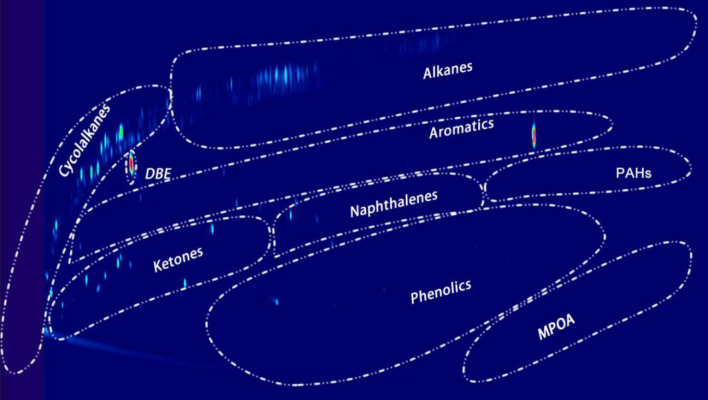
Liquid product analysis with GC-GC (reaction was performed with Pt/C as the catalyst, the reaction temperature was set at 400 °C, and reaction time was 7 h). DBE was the internal standard for GC analysis.

These chemical classes were detected again by GCxGC (Fig. 22), which is a new technique to analyze organic compound classes present in bio-oil because they will be shown in special regions in the GCxGC figures. Results from GC–MS and GCxGC were the same.

**Figure 23:**
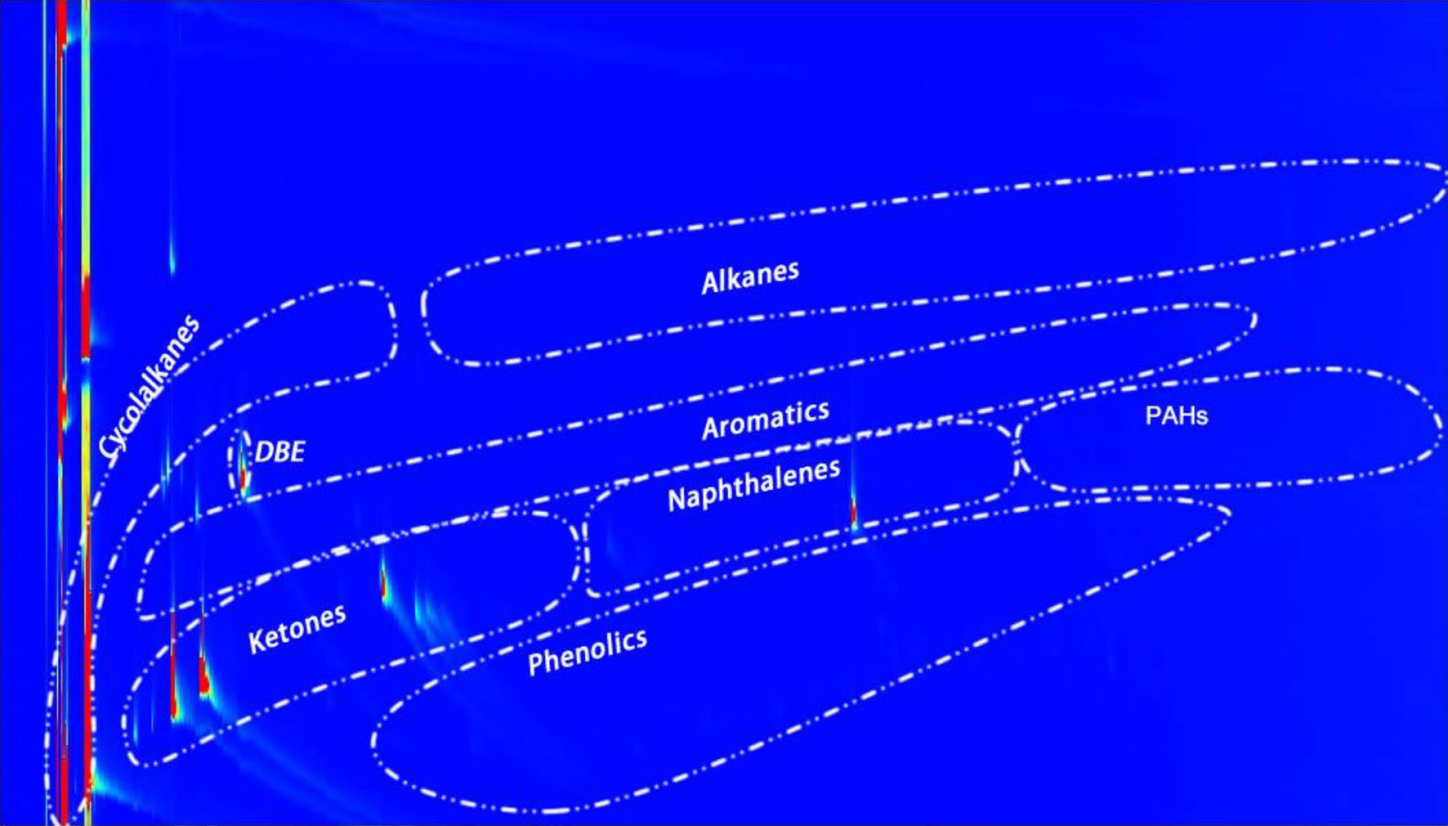
GCxGC plot of the liquid phase of a blank reaction.

**Figure 24:**
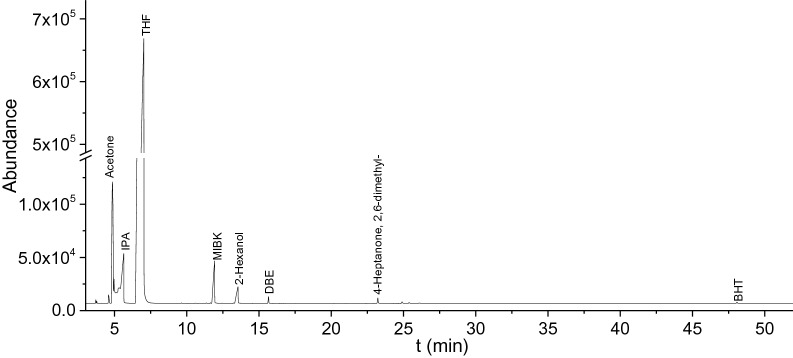
GC–MS-FID results of blank reaction (Pt on carbon as the catalyst, reaction temperature is 400 °C and time was 7 h with THF as the solvent, DBE is used as the internal standard for the analysis and BHT is the stabilizer of THF).

IPA is active under reaction conditions because we had found there were gas phase components formation in the blank reaction (only IPA and catalyst, Figs. 23, 24). Acetone, methylsiobutylketone (MIBK), 2-hexanol, and 2,6-dimethyl-4-heptanone were observed in the liquid phase.

**Scheme 1.**
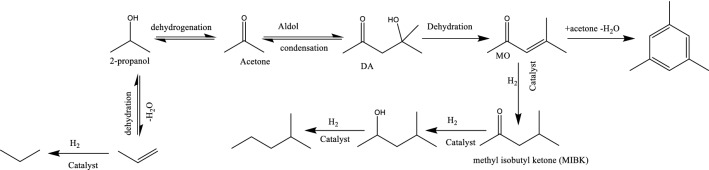
Possible reaction pathway for IPA (400 °C, Pt/C).

It is possible that ketones and alcohols were produced from IPA (Scheme 1). The process included dehydrogenation of IPA to acetone, aldol condensation to diacetylacetone (DA), mestityleoxide (MO) formed from DA, and finally catalytically hydrogenated to MIBK. Under the reaction conditions with Pt, ketones could be hydrogenated to produce the related alcohols. These reactivities of IPA had been reported by Kleinert et al. 18 and Kloekhorst et al. 32.

**Figure 25:**
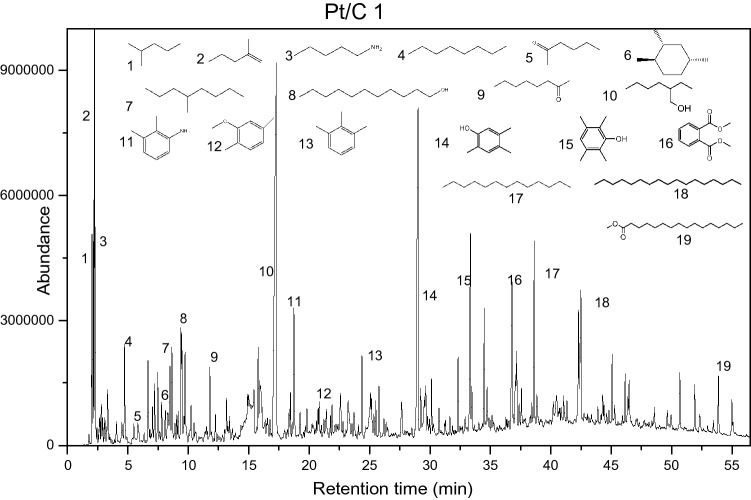
gc–ms analysis of liquid product using Pt/C 1.

**Figure 26:**
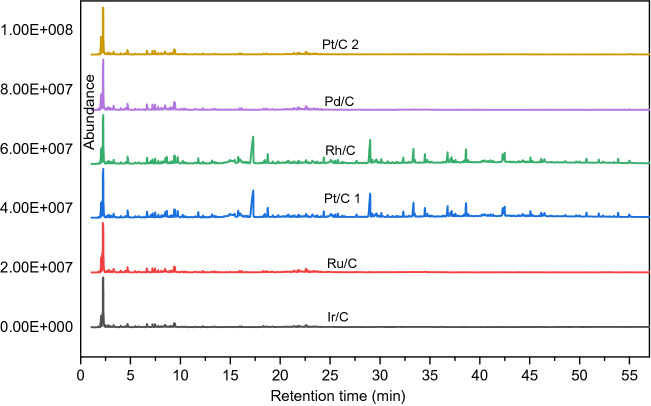
Gc–ms peaks of liquid products with different catalysts.

Effect of catalyst on the liquid substances:

Gc–ms analysis was performed on all liquid product from different catalyst, and the relevant data analysis is shown in Figs. 25, 26 and Fig. S4 to Fig. S8 in the supporting information.

**Figure 27:**
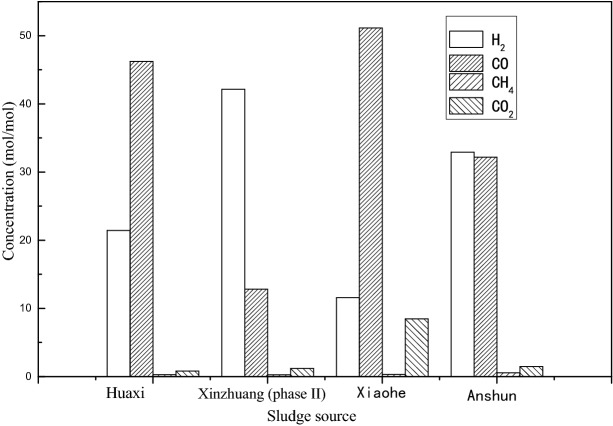
Gas phase composition and concentration diagram of sludge pyrolysis.

It is known from Fig. 27 that the liquid phase products using Ir/C, Ru/C, Pd/C, and Pt/C 2 catalysts, the peak trends of the substances are almost the same (group a), while peaks from Pt/C 1 and rhodium/C were almost similar (group b), and group b has more peaks than group a, and the peak height and peak area are bigger than group a. These results showed that under the same conditions, the liquid phase products of Pt/C1, Rh/C had more contents and product types than the liquid phase products from Ir/C, Ru/C, Pd/C, Pt/C2.

Effect of solvent on the liquid substances:

The liquid products from differerent solvents were analyzed by gc–ms and the results were shown below:

1. Some small molecules in the liquid phase from isopropanol as solvent are:

Formaldehyde n-butyl formate; 2,4,6-trimethylpyridine; 2,4-di-1,1-dimethylethylphenol; 3,5-cyclohexadiene-1,2-dione- 3,5-Di-1,1-dimethylethyl; 1-bromoethyl-4fluorobenzene; 2,2-dimethyl-3-hexanone; malonamide; 2- (2-hydroxyethyl (Oxy) ethyl-octadecanoic acid; trimethylamine oxide; trimethylisopyrazine; 3,5-dimethylcyclohexanone; 2,3-dimethyl-1,4-pentene-2- Alcohol; 1,2-dimethylcyclohexane; 1,3-butadiene-1-carboxylic acid; thienyl chloride; 2-acetyl-1-phenylhydrazine; 1,1,1-trinitroethane ; 1,3,5-triazine-2,4,6-triamine; 2 (3 h) -furanone, dihydro-5-methyl; n-ethane-nn-dimethyl-n- Nitrosourea; 2,4-di-1-methylethyl-phenol.

2. Some small molecules in the liquid phase from ultrapure water as solvent are:

Bromoethyl mercury; 1-acetaldehyde–2,3,6-trichlorobenzene; 1-n-butylamine-n-butadiene; hexene; methyl succinic anhydride; 2-methyl-propanal; Chloro-acetic acid; 1,1-ethylene-di-oxy-di-propane; isopropanol; dextroamphetamine; butyl mesylate.

3. Some small molecules in the liquid phase from isopropanol and formic acid as solvents are:

Acetone; n-pentane; pyroglutamic acid; 3-methyl-pentane; 3-butyn-2-ol; hexene; methyl 2-butyrate; dl-3-methylcyclopentanone; methyl Cyclohexane; cycloheptane; dl-camphorquinone; dimethyl phosphite; 2-aminopyridone; 2-acetyl-5-methylfuran; 1,2-dimethyl-cyclohexane; 1,5 -Dimethyl-2-pyrrolidone.

4. Some small molecules in the liquid phase from isopropanol and methanol as solvents are:

D-amphetamine; benzoylmethylsulfonyl chloride; n- (2-methoxyphenyl) -3-oxo-butyramide.

5. Some small molecules in the liquid phase from isopropanol and ethanol as solvents are:

1,1-ethylidene diphosphine-di-oxy-propane; alkylbenzene; cycloheptane; 2,5-dimethylfuran; 2,5-dihydropyrrole; methyl caprylic acid; DL-camphorquinone; 3 , 4-dimethyl-2-hexanone; pyrrolidine; n-pentanol; 2,2-diethoxypropane; 1,2-dimethyl-cyclohexane; vinyl-ethylene oxide; 2 (1H) -pyridone; 2-ethoxy-ethanol; 1,4-cyclohexanedione; butyric acid; 2-ethane-2-butenal; 6-hydroxy-2 (1 h) pyridone; Methyl bromide; 3,5-dimethylcyclohexanone.

We can conclude that solvent has a strong effect on the liquid products if the others conditions are same, so solvent should be taken in account if more researches will be performed in this research aerea.

### Characterization of gas products

#### Effect of source of sewage sludge to gas products

The terminal pressures of sludge pyrolysis reactions from different sewage sludge source were shown Table 19:

**Table 19:** Terminal pressure of sludge pyrolysis reaction.

Huaxi sewage treatment plant (MPa)	Xinzhuang wastewater treatment plant (phase 1) (MPa)	Xinzhuang wastewater treatment plant (phase II) (MPa)	Xiaohe sewage treatment plant (MPa)	Anshun green power renewable energy Co (MPa)
1.9	1.7	1.8	2.0	2.0

It is known that the terminal pressure is almost same in all reactions at same conditions, which meant that the total amount of gas products might be almost same although their compositions might be different.

External standard quantitative analysis method was applyied to the gas-gc measurement of gas product from the pyrosis of sewage sludge, and the calculation formula is:

$$ C_{i} = \frac{{C_{s,i} }}{{A_{s,i} }} * A_{i} $$

where *C*_*i*_ , in the sample $i$ content of components; $C_{s,i}$, in the standard sample $i$ content of components; $A_{s,i}$, in the standard sample $i$ peak area of the component; $A_{i}$, in the sample $i$ peak area of the component;

The related data of standard gas are shown in the Table 20.

**Table 20:** Standard gas peaks.

Component	Content (mol/mol) (%)	Peak time (5 min/group)
H_2_	15.0	0.3–0.5 min
CO	6.01	0.7–0.9 min
CH_4_	4.00	1.9–2.2 min
CO_2_	5.02	3.0–3.6 min
Ar	69.97	–

The gas product results of sludge pyrolysis obtained by calculation are shown in Table 21.

**Table 21:** Summary of gas product distribution from pyrolysis of sewage sludge.

Gas type (mol/mol)	H_2_	CO	CH_4_	CO_2_	Total concentration
Huaxi sewage treatment plant	21.44	46.24	0.29	0.82	68.79
Xinzhuang wastewater treatment plant (phase 1)	–	–	–	–	–
Xinzhuang wastewater treatment plant (phase II)	42.15	12.83	0.28	1.20	56.46
Xiaohe sewage treatment plant	11.58	51.15	0.31	8.47	71.51
Anshun green power waste power plant	32.92	32.20	0.57	1.48	67.17

The gas composition analysis results of sludge pyrolysis from different sources also were shown in Fig. 27.

The following conclusions could be obtained from Fig. 27 and Table 20:

1. The yields of methane and carbon dioxide in the catalytic hydropyrolysis gas of sewage sludge from different sewage treatment plants are the least.
2. Carbon monoxide keeps the largest amount in all sources except for the Xinzhuang Wastewater Treatment Plant (Phase II). According to the FTIR data analysis of sludge from various sewage treatment plants, it can be inferred that carbon monoxide comes from the carbonyl groups, primary fatty alcohols and secondary fatty alcohols in the sewage sludge.
3. The amount of carbon monoxide produced by the dewatered sludge in the Xiaohe Sewage Treatment Plant is much larger than the sludge samples of the other 3 sewage treatment plants under the same experimental conditions. Polyacrylamide, which contains carbonyl groups, will increase output.
4. Due to the limitation of instrument detection technology, only five gases, including hydrogen, carbon dioxide, carbon monoxide, methane and argon had be detected.
5. After the pyrolysis of sludge from different sewage treatment plants, more carbon monoxide gas is generated because carbon monoxide is the basic raw material for the production of carbonyl chemicals^103^. Therefore, carbon monoxide in the sludge pyrolysis gas can be separated as a resource choice of sludge pyrolysis gas.
6. The sludge pyrolysis gas contains hydrogen, which may come from the preparation of the reaction system, or it may come from the cracking of sludge and isopropanol, so it should be distinguished whether the hydrogen comes from the sludge pyrolysis.

#### Effect of catalyst to gas products

The terminal pressures of sludge pyrolysis reactions from different sewage sludge source were shown Table 22:

**Table 22:** Pressure at the end of the experiment.

Catalyst	Pressure (MPa)
Ir/C	1.9
Ru/C	1.9
Pt/C 1	1.9
Rh/C	1.7
Pd/C	1.9
Pt/C 2	1.7

It is known that the terminal pressure is almost same in all reactions at same conditions, which meant that the total amount of gas products might be almost same although their compositions might be different.

The calculation and analysis methods were same to the former part, and here the content of the gas products was shown. The results are shown in Table 23.

**Table 23:** Components and contents of gas-phase products.

Catalyst	c H_2_ (%)	c CO (%)	c CH_4_ (%)	c CO_2_ (%)	Total concentration	H_2_/CO
Ir/C	7.57	62.14	0.21	0.87	70.79	8.21
Ru/C	19.59	49.02	0.27	0.87	69.75	2.50
Pt/C 1	9.49	54.35	0.26	0.79	64.88	5.73
Rh/C	29.51	25.00	0.45	1.10	56.06	0.85
Pd/C	46.94	10.35	0.45	1.15	58.88	0.22
Pt/C 2	53.76	10.95	0.45	1.01	66.17	0.20

**Figure 28:**
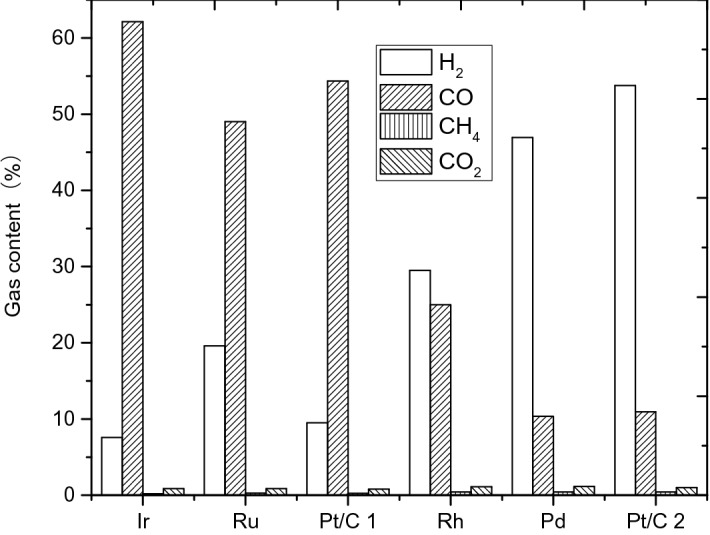
Composition and content of gas phase products.

The comparison of the percentage change of the concentration of each component also was shown in Fig. 28.

Based on these analysis, we can see that the partial pressures of H_2_ in the gas phase products from Ir/C, Ru/C, Pt/C 1, Rh/C, Pd/C, and Pt/C 2 are 0.144 MPa , 0.372 MPa, 0.180 MPa, 0.502 MPa, 0.892 MPa, 0.94 MPa, respectively. It is known that the pressure of hydrogen is lower than 1 MPa, which is the start hydrogen pressure, indicating that hydrogen may not be the experimental product. This conclusion also could be proved from the fact that the order of hydrogenation capacity of these catalysts is: Ir/C > Pt/C1 > Ru/C > Rh/C > Pd/C > Pt/C2.

#### Effect of solvents to gas products

The calculation and analysis methods were same to the former part, and here the mole fraction of the gas products was used. The results are shown in Table 24.

**Table 24:** Summary of gas product concentration from different solvents(unit: mol/mol).

Solvent	H_2_	CO	CH_4_	CO_2_
Isopropanol	15.89	54.59	0.24	0.96
Ultra-pure water	37.50	8.28	4.70	2.73
Isopropanol + formic acid	55.43	12.45	0.31	3.70
Isopropanol + methanol	28.68	40.27	0.83	0.83
Isopropanol + absolute ethanol	41.29	9.43	0.33	2.38

**Figure 29:**
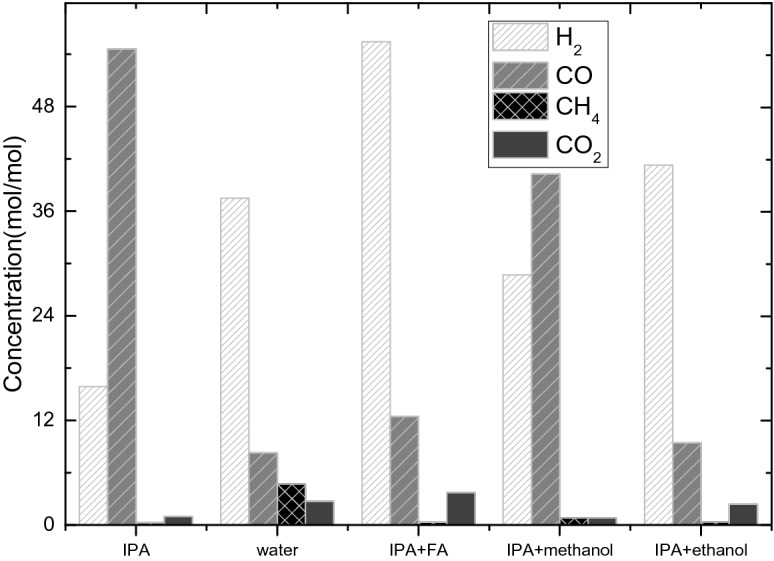
Composition of different components in the gas product.

The results also were shown in Fig. 29.

It can be known from Table 24 that for isopropanol as the solvent, the hydrogen and methane are the least compared to the other four solvents, only 15.89% and 0.24%, but the carbon monoxide (54.59%) is the most. This is because Isopropanol contains three carbon atoms. Compared with other solvents, carbon dioxide has the most carbon atoms. Under high temperature conditions, carbon and water react to form carbon monoxide and hydrogen. When isopropanol was used, the solvent did not contain water, and the moisture is small, so the amount of hydrogen produced is small.

As can be seen from Table 24, methane (4.70%) produced more when ultrapure water was used as the solvent, because water molecules are polar molecules, and more hydrogen ions are dissociated than other solvents. The carbon atoms in methane are derived from the decomposition of organic matter in the sludge. There may be carbon–carbon double bond breaks and carbon–carbon single bond breaks. Methane requires four hydrogen ions, so more hydrogen ions are generated for dissociation.

Formic acid (hcooh) is known to decompose in the presence of a ruthenium-carbon catalyst to produce carbon monoxide, carbon dioxide and hydrogen^16^. Under different conditions, the decomposition products of formic acid are different. When aerobic participation occurs, the dehydration reaction occurs and the products are carbon monoxide and water. In this experiment, the reaction conditions were high temperature with hydrogen afford and Ru/C was used as catalyst, and Under the catalytic action of formic acid, the decomposition reaction of formic acid occurs at 220–280 °C and generate hydrogen and carbon dioxide. Therefore, in the five solvent conditions of this experiment, the reaction using isopropanol and formic acid as the solvent produced more hydrogen and carbon dioxide than others four solvents. The hydrogen content is 55.43% and the carbon dioxide content is 3.70%.

Methanol and sludge could react under high temperature conditions, and the by-products are methane and carbon oxides, but from the table we can see that the solvent produced the most methane (0.83%) and carbon dioxide (CO: 40.27%; CO_2_ 0.83%) was not methanol. One reason is that the gas which was used to flush the reactor was hydrogen, and hydrogen is one product of methanol decomposition, so a reaction system with equal amounts of products has not been formed, thereby consuming a part of the oxygen compounds; The second reason is that if there is only methanol in the reactor, methanol will react near the active center of the catalyst.Because a large amount of reaction heat is generated, the temperature of the place where the reaction occurs is too high, causing the actual temperature to be higher than the detected temperature, and these local heat will lead to faster deactivation of the catalyst coke, which will destroy the stability of the catalyst.Therefore, if the amount of methanol in the reaction system is generated in turn, the catalytic performance of the catalyst will be reduced first, resulting in an increase in the content of by-products and methane gas. As a result, it has increased.

For the experiments with the ethanol as a part of the solvents, the gas produced was relatively small, and the hydrogen (41.29%) was only less than the formic acid. The reason is that organic matter may be decomposed to produce hydrogen during the catalytic cracking of sludge. And it is not certain whether the hydrogen is a product or the left hydrogen which was use to flush the reactor.

## Conclusion

Catalysts have a obviously effect on the catalytic hydrogenation of sewage sludge , and catalytic effect of Ir/C, Pt/C 1, Ru/C, Pd/C or Pt/C c are relatively high.

Pt/C 1 catalyst has a better effect on the catalytic hydropyrolysis of sewage sludge, and the gas phase product using the Pt/C 1 catalyst contains a large amount of co, which can be used as a reducing agent, such as Industrial reduced iron oxide (prepared iron) $(CO + F_{e2} O_{3} = 2F_{e} + 3CO_{2} )$. Methanol is a chemical raw material and can also be used to produce acetic acid. It is often used as an organic extractant and analytical reagent (solvent, chromatographic analysis reagent). It is a better solvent than ethanol, it can dissolve many inorganic salts, and can be blended into gasoline Used as an alternative fuel. Methanol has also become a new fuel for the twenty-first century. It is easily pressurized into a liquid and easy to store. It is a new liquid fuel (alcohol-ether fuel) with a certain proportion of dimethyl ether. It has both combustion efficiency and thermal efficiency. It is higher than liquefied gas. Therefore, it can also be used to produce methanol, which will help improve the resource efficiency of sludge and achieve indirect resource utilization of sludge.

Based on the above conditions, in this experimental study, the sludge catalytic hydrogenation performance is good, and the Pt/C 1 catalyst is used to produce valuable small molecules with a high yield.

The substances in the solid phase of sludge pyrolysis are mainly inorganic phosphates and fatty alcohols. The gas products after sludge pyrolysis are mostly CO, CO_2_, CH_4_, and the main components in the sludge pyrolysis liquid phase are some small organic molecules: alcohols, esters, alkanes, olefins, furans, pyrimidines, carboxylic acids, furoic acids, and ketones.

## Supplementary information

Supplementary Information
