# Supplementary material for: Catalytic liquefaction of sewage sludge to small molecular weight chemicals
Source: Sci Rep. 2020 Nov 3;10:18929. doi: 10.1038/s41598-020-75980-z (PMC7609695; doi:10.1038/s41598-020-75980-z)

Catalytic Liquefaction of Sewage Sludge to Small Molecular Weight Chemicals

Yuehu Wang[*1,2], Feihong Tian[1,2], Peimei Guo[1,2], Dazhen Fu[1,2], Hero Jan Heeres[3], Taotao Tang[1,2]. Huayu Yuan[1,2], Bing Wang[1,2], Jiang Li[1,2]

[1]College of R esources and Environmental Engineering， Guizhou University， Guiyang， 550025， China;

[2]Observation and R esearch Station for Guizhou Karst Environmental Ecosystems， Guiyang， 550025， China

[3]Chemical Engineering Department, ENTEG, University of Groningen, Nijenborg 4, 9747 AG Groningen, The Netherlands

* Corresponding author: wangyuehu814081@163.com

For publication in: Scientific Reports

Supplementary material.

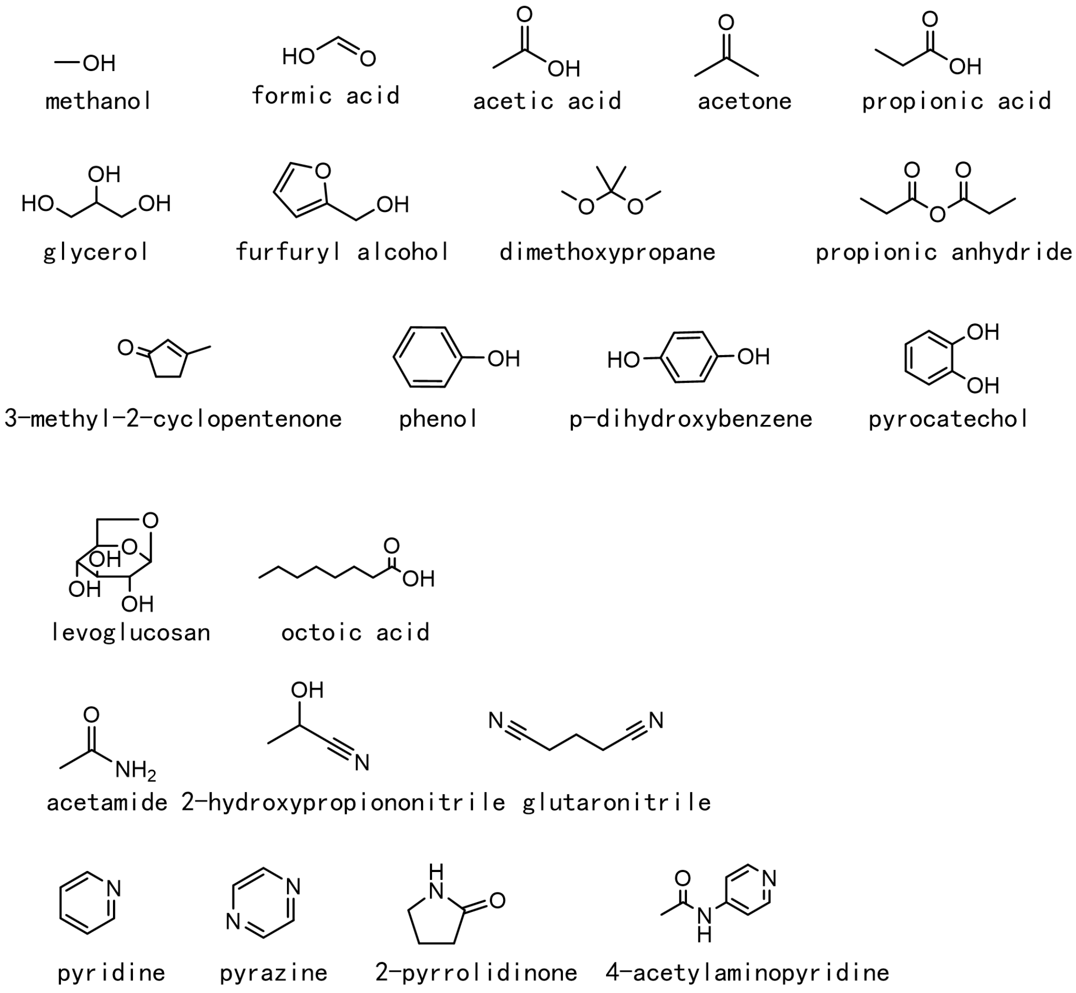

Fig. S 1 possible pyrolysis products of sewage sludge.

Huaxi sewage treatment factory: Huaxi sewage treatment factory locates in the Wenyan village,Jingkai district, Guiyang city(106.705442°E, 26.454704°N), and which cover an area of 93.45 mu.Total of phase 1 and phase 2 have a design water treatment quantity of 40 thousand m^3/d, and the total design scale is 80 thousand m^3/d. the investment assets of phase 1 is 120 million yuan, and the investment assets of phase 2 is 80 million yuan. the sewage treatment adopts sequencing batch reactor activated sludge process (SBR), and its process flow chart was shown in Fig S 2.

It was run in november 2004, and it was upgraded in december 2014 to make the exceed water quality elevate from level IB to level IA (pollutant discharge standard of urban sewage treatment plant in China), and its exceed water is discharged into the nearby Chengliang river.biochemical treatment of sewage adopt CAST technology,advanced sewage treatment adopts biological active sand filter and high efficiency settler, and disinfection of effluent use chlorine dioxide, which was put into run in January 2015.Its mostly water quality index of effluent reach to surface water IV water quality standard.The others water quality index execute the level IA standard of 'pollutant discharge standard of urban sewage treatment plant' （G818918-2002）. Its main service area includes in the Huaxi town south, Yangniu, Luoping, Huaxi town north, Xiao huanghe south and

Chengliang area. Its service population is about 620 thousand.

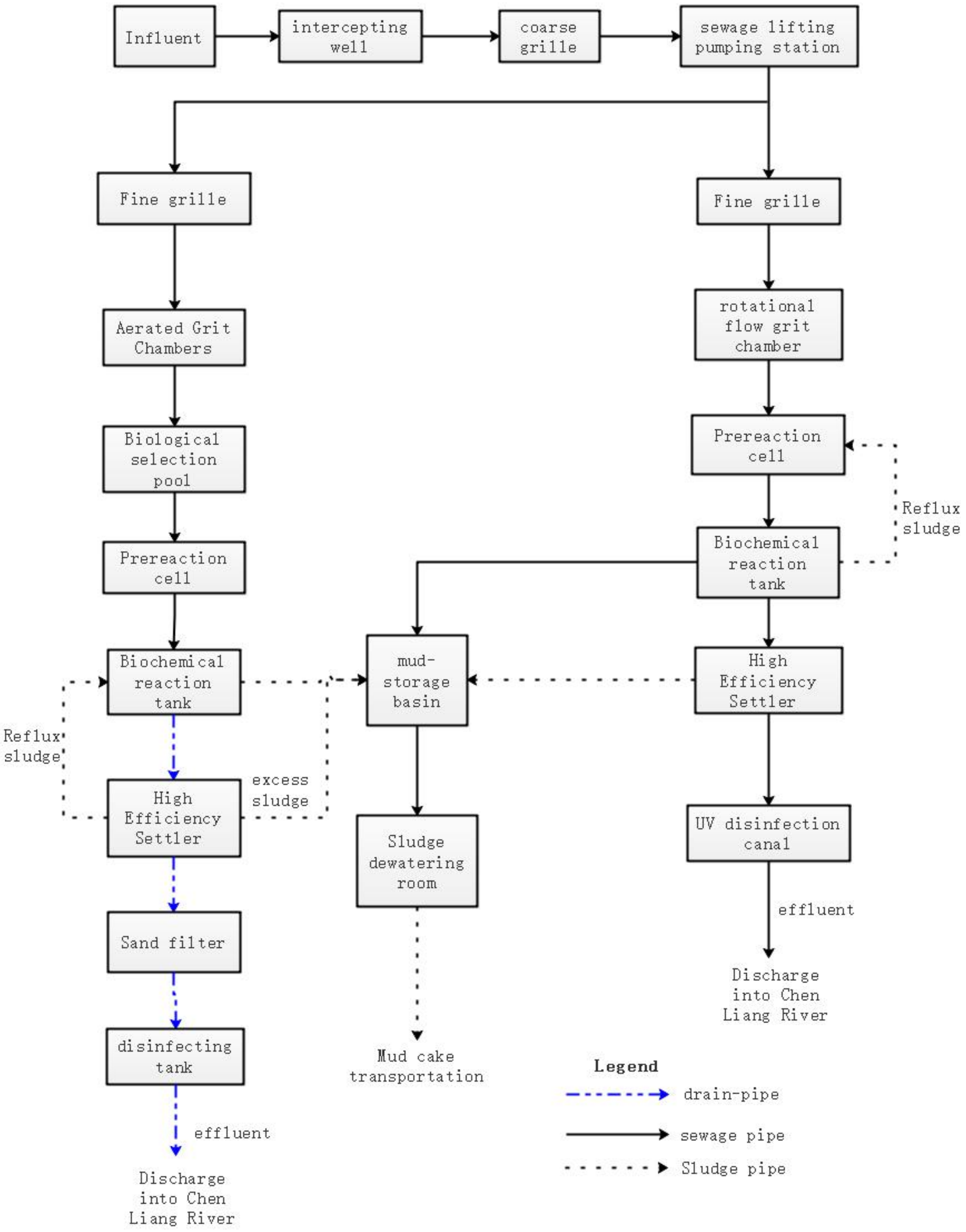

Fig. S 2 Process flow chart of Huaxi Sewage Treatment Plant

The standard operation process is: (1) Put the weighed samples into the reaction kettle in turn, and the stakeout order is catalyst, sludge, and solvent; (2)Close the kettle, tighten the reaction kettle and the intake valve bolts diagonally, close the intake valve and exhaust valve;(3)Start the reaction kettle controller; (4)Open the the successive pressure reducing valve of nitrogen tank and its control valve to keeep the reactor and pipes with 1MPa nitrogen and maintain the pressure for 15min; (5) Check whether the reactor is closed properly, that is, check the air tightness, whether there is a leak, if the pressure is being maintained, If the pressure decreases, it means that the air tightness of the reaction kettle is not good; (6) After 15 minutes, flush the air in the kettle three times with nitrogen; (7)After flushing, close the reaction kettle valve, nitrogen tank control valve, nitrogen tank valve and pressure reducing valve in turn; (8)Open the hydrogen tank Valve, open

the hydrogen tank pressure reducing valve and control valve in sequence, access hydrogen into the reactor which was filled with nitrogen; (9)Wash the reactor twice with hydrogen; (10)After keep the pressure to 1 MPa three times, close the inlet valve, control valve, hydrogen tank valve and pressure reducing valve in turn; and then (11)Set the controller parameters:

C01:15℃，TO1:72min；

CO2:360℃, TO2:180min;

CO3:360℃, TO3:40min;

CO4,15℃, TO4: -121.

The speed is set at 600 rpm.

(12) Press the ‘run’ key for two seconds to start running, and record the maximum pressure and temperature during the reaction is running.

(13) After the temperature has dropped to the set temperature, record the final pressure and temperature on the controller, and use a gas bag to collect the gas generated after the reaction finished; (14) Open the air release valve and use the gas bag to collect the gas after removing the air in the pipe; (15) After the gas is collected, open the gas release valve to release all the gas inside, and then open the kettle. The centrifuge tube used is weighed in advance; (16) After open the kettle, use a plastic tip dropper to suck the reacted materials in the reaction kettle into a 50ml centrifuge tube. Record the total weight and prepare two additional centrifuge tubes to add water with same weight; (17) Put three centrifuge tubes into the centrifuge and place them diagonally to maintain balance, set the speed to 5000 rpm for 30 minutes; (18)Remove the centrifuged sample, and pour the supernatant after centrifugation into a 20ml sample bottle; (19)Put the precipitated part of the sample after centrifugation into an electric blast drying oven of model 101-3AB; (20) Set the temperature at 75 ℃ for 4 hours, remove the dried precipitate, weigh it, and record the weight.

Analytical method:

For the catalysts used in the experiments were characterized with sem, ftir and tga. Gas phase products was analyzed with gas-gc to investigate the qualitative and semi-quantitative composition of catalytic cracking products. The solids and liquids collected after the experiment were sent to the Guizhou Provincial Analysis and Testing Institute and the University of Groningen for analysis. The solids were analyzed by tga, sem and ftir; the liquids were analyzed by gpc, gc-ms, 2D-GC-MS/FID (University of Groningen). The technologies were explained simply below:

tga: thermogravimetric analyzer, which can keep the sample at a program-controlled temperature and observe the function of the sample's mass as a function of temperature or time. Through these changes, we can get information about the thermal stability and composition of the material, usually the curve change in milligrams, or as a percentage change in starting weight;

sem: scanning electron microscope, a microscopic morphology observation method between projection electron microscope and optical microscope, which mainly uses secondary electronic signals to directly observe the structure of the sample surface;

ftir: Fourier transform infrared spectroscopic analysis, which can detect a variety of different chemical molecules;

gpc: gel permeation chromatography, also known as size exclusion chromatography, is used to separate substances with relatively small molecular mass.It can also analyze polymer homologs

with different molecular volumes and the same chemical properties, measure the molecular weight distribution of polymers, and detect whether the sample monomer has polymerized;

gc-ms: gas chromatography mass spectrometer, which is a gc and ms integrated device, and it is a qualitative and quantitative detection method for chemical compounds.

More information for some chemicals:

1.Isopropanol: molecular formula $(CH_3)_2CHOH$, manufactured by Tianjin Fuyu Fine Chemical Co., Ltd., conforms to the isopropanol industry standard. Conforms to: hg/t2892-2010 production standards. Transparent liquid. It has a slight smell of ethanol, is miscible with water, alcohol, and ether, and is highly flammable.

Maximum impurity content (indicated by ≤%)

Evaporation residue, w%-≤ 0.001

Mixing experiment with water-qualified

Reduction of potassium permanganate substance-qualified

Methanol (CH_3OH) w%-≤ 0.1

Iron (Fe) W% —— ≤0.00001

Easily carbonized substances-qualified

Carbonyl compound (based on co) w% ---- ≤0.005

Moisture (H_2O) w%-≤0.2

Acidity (as H +) mmol/g——≤0.003

2.Methanol: molecular formula CH_3OH, manufactured by Tianjin Fuyu Fine Chemical Co., Ltd., conforms to the production standard of methanol national standard: gb/t683-2006, the reagent used is a colorless transparent liquid, which can be used with water, ether, alcohol, etc. Miscible, vapor pressure and air can form an explosive mixture, which generates a blue flame when burning.

Maximum impurity content (indicated by ≤%)

Mixing experiment with water-qualified

Moisture (H_2O)-0.1

Reduction of potassium permanganate substances-≤ 0.0005

Carbonyl compound (based on CO)-≤ 0.005

Evaporation residue——≤0.001

Easily carbonized substances-qualified

Acidity (as H^+) mmol/g——≤0.0004

Alkalinity (calculated as OH^-) mmol/g——≤0.00008

3.Formic acid: molecular formula HCOOH, manufactured by Chengdu Jinshan Chemical Reagent Co., Ltd., in accordance with the production standard of formic acid GB: gb/t15896-1995, the reagent used is a colorless transparent liquid with irritating odor, flammable, corrosive, and Mix water, ether, alcohol, glycerin, specific gravity 1.22γ 204, and formic acid content not less than 88.0%.

Maximum impurity content (in%)

Evaporation residue-0.002

Mixing experiment with water-qualified

Chloride (Cl)-0.0005

Iron (Fe)-0.0003

Heavy metals (as Pb)-0.0003

Sulfate (SO_4)-0.001
Sulfite (SO_3)-qualified
4.Anhydrous ethanol: molecular formula CH_3CH_2OH, manufactured by Tianjin Fuyu Fine Chemical Co., Ltd., in compliance with the national standard: gb/t678-2002 production standard, the reagent used is a colorless transparent volatile liquid, It has a strong fragrance and is easy to absorb moisture.It can be mixed with water, ether and chloroform at will.It is highly flammable and the vapor can form an explosive mixture with air.
Technical index
Mixing experiment with water-qualified
Mass fraction of evaporation residue——≤0.001
Mass fraction of methanol (CH_3OH) % ≤ 0.05
Mass fraction of isopropanol ($(CH_3)_2CHOH$) %-0.01
Mass fraction of carbonyl compound (based on CO) % ≤ 0.003
Acidity (as H^+) mmol/100g——≤0.04
Alkalinity (based on OH^-) mmol/100g——≤0.01
Mass fraction of water% ≤ 0.3
Easily carbonized substances-qualified
Mass fraction of reduced potassium permanganate substance-≤ 0.00025
5.Ultra-pure water: Produced by Yiliyuan 151 laboratory ultra-pure water machine yl-100bd, and purified by activated carbon + ultrafiltration, pPF cotton, ro membrane reverse osmosis filtration.

Table S 1 Main technical parameters of the reactor

index	Nominal volume l					
project	0.1-0.5	1	2	3	5	10-30
Working pressure MPa	≤35.0MPa					
Operating temperature	≤350℃					
heating method	Electricity, steam, thermal oil, far infrared, water cycle, etc.					
Heating power kw	1	1.5	1.5	2-3		6
Stirring speed rpm	20-800rpm adjustable					
Motor power w	92	123	185	245	355	550-750
Body material selection	Various grades of austenitic stainless steels 321, 304, 316l, etc., titanium, nickel, zirconium, tantalum, Hastelloy, PTFE, etc.					
Controller	With self-tuning function, intelligent digital temperature control meter, with automatic constant temperature and temperature control functions, with speed display and stepless speed adjustment functions, equipped with heating voltmeter, motor ammeter, working time display meter, and according to user requirements Equipped with Xiamen Yuguang intelligent program heating meter, matching computer interface and related software, as well as remote pressure overpressure alarm and other functions.					
Controller working environment	The ambient temperature is 0-50 ℃, the relative humidity is 30-85%, and the surrounding medium does not contain conductive dust and corrosive gases.					

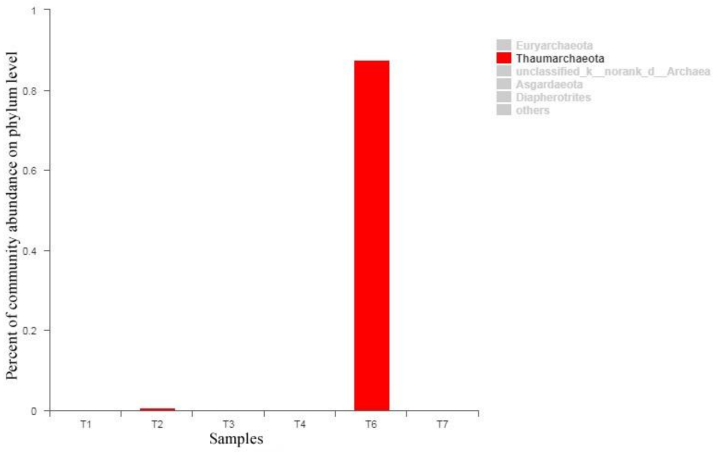

Fig. S 2 Proportion of Thaumarchaeota abundance in purified samples

Table S 2 Summary of functional groups in sludge

Factory name	Functional group
Huaxi Sewage Treatment Plant	Primary aliphatic amides, inorganic phosphates, primary aliphatic alcohols.
Xinzhuang Wastewater Treatment Plant (Phase 1)	Inorganic carbonate, inorganic phosphates, primary aliphatic alcohols.
Xinzhuang Wastewater Treatment Plant (Phase II)	Inorganic carbonate, inorganic phosphates, primary aliphatic alcohols.
Xiaohe Sewage Treatment Plant	Inorganic phosphates, aliphatic secondary amides, primary aliphatic alcohols.
Anshun Green Power Waste Power Plant	Inorganic nitrites, primary aliphatic amides.

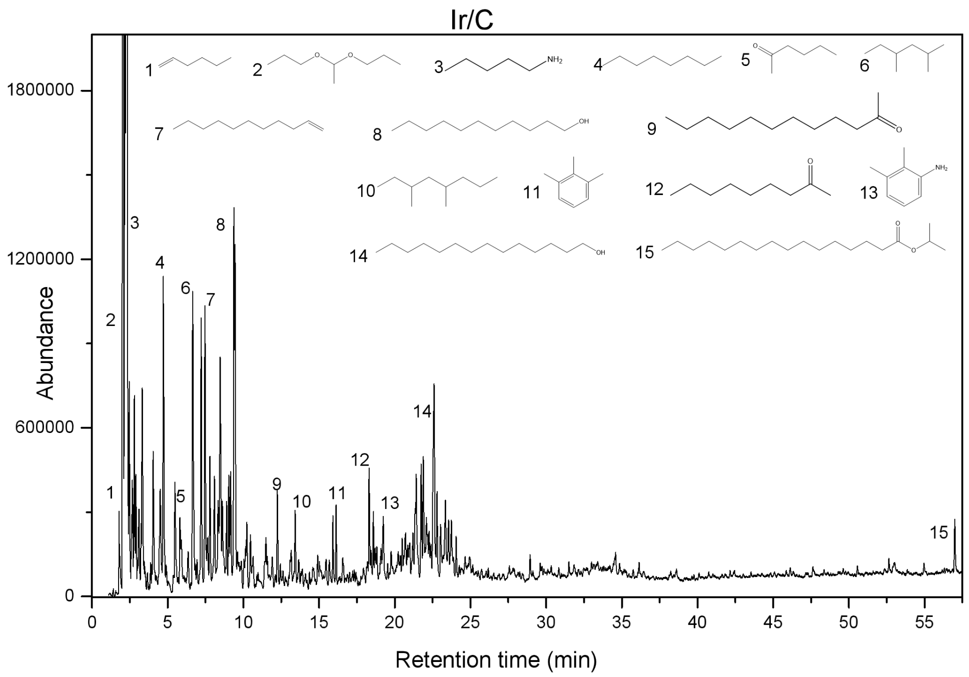

Fig. S 3 gc-ms analysis of liquid product using Ir/C catalyst

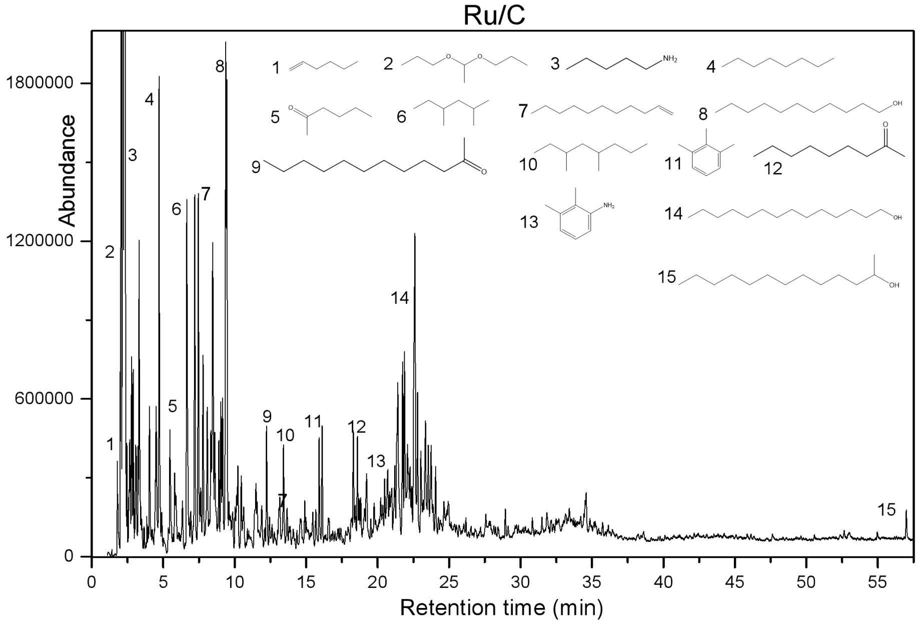

Fig. S 4 gc-ms analysis of liquid product using Ru/C catalyst

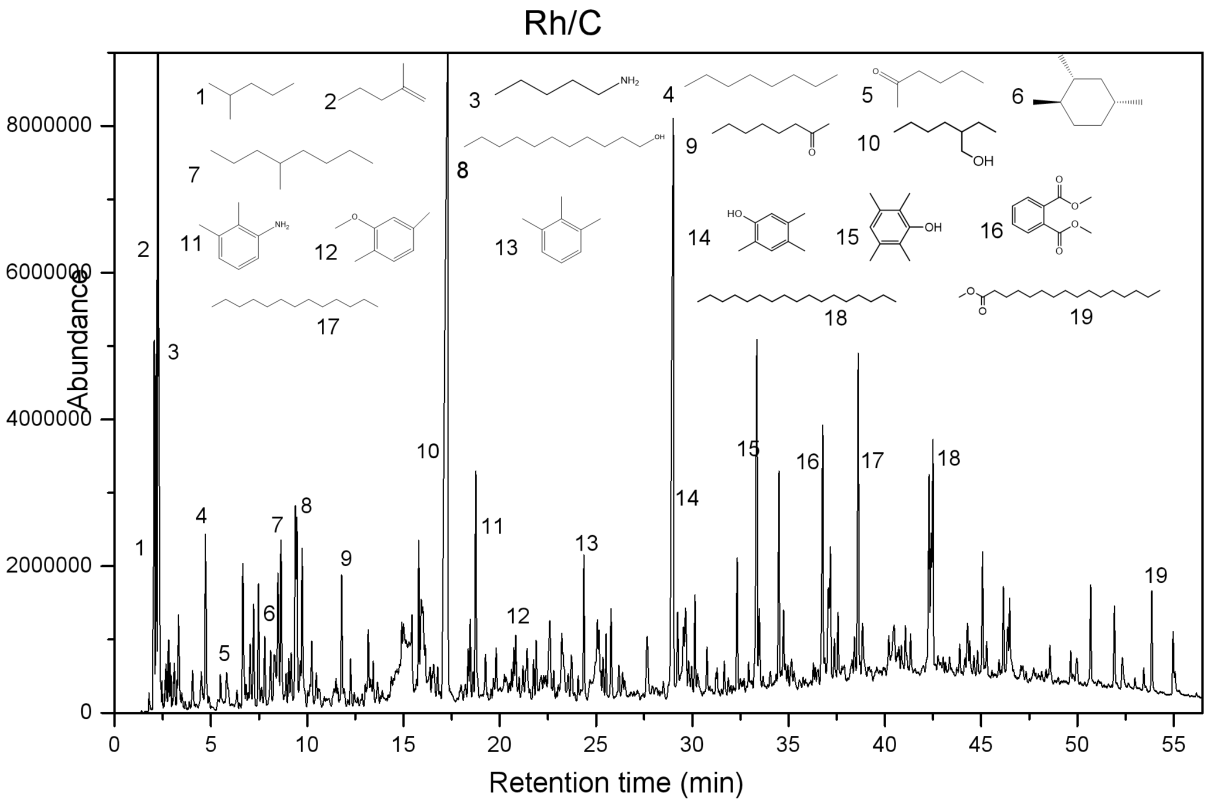

Fig. S 5 gc-ms analysis of liquid products catalyzed by Rh/C

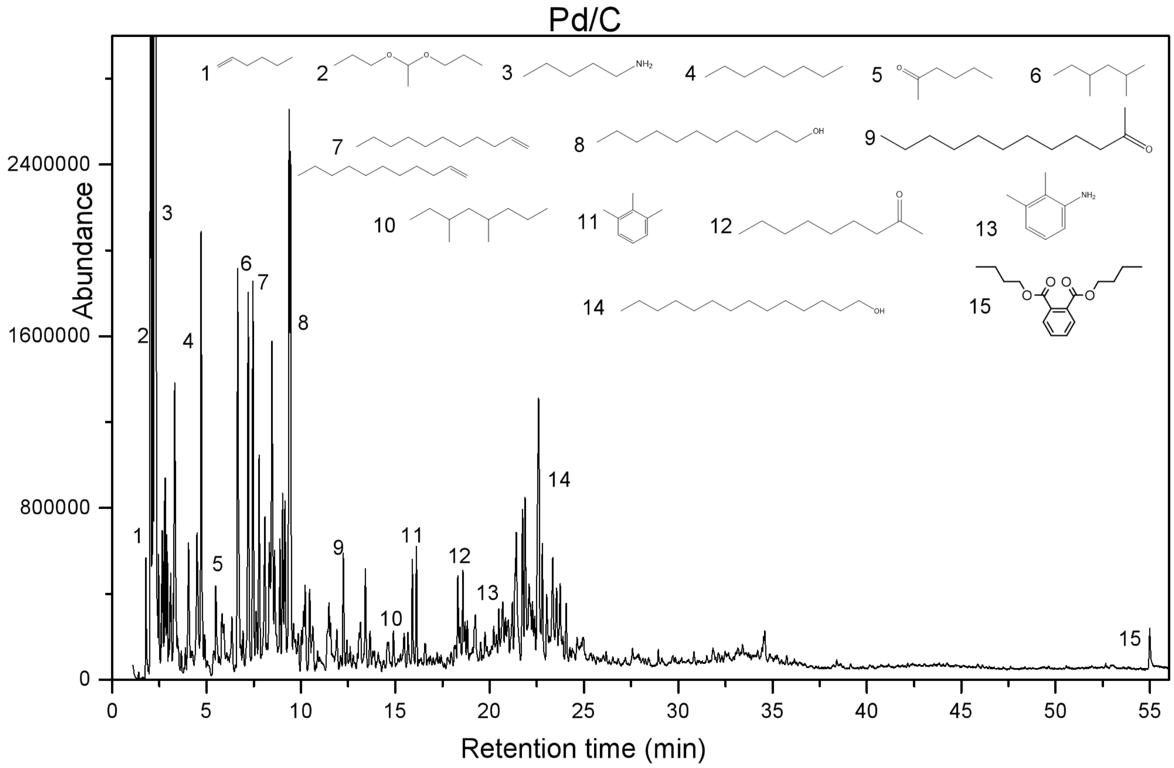

Fig. S 6 gc-ms analysis of liquid product using Pd/C catalyst

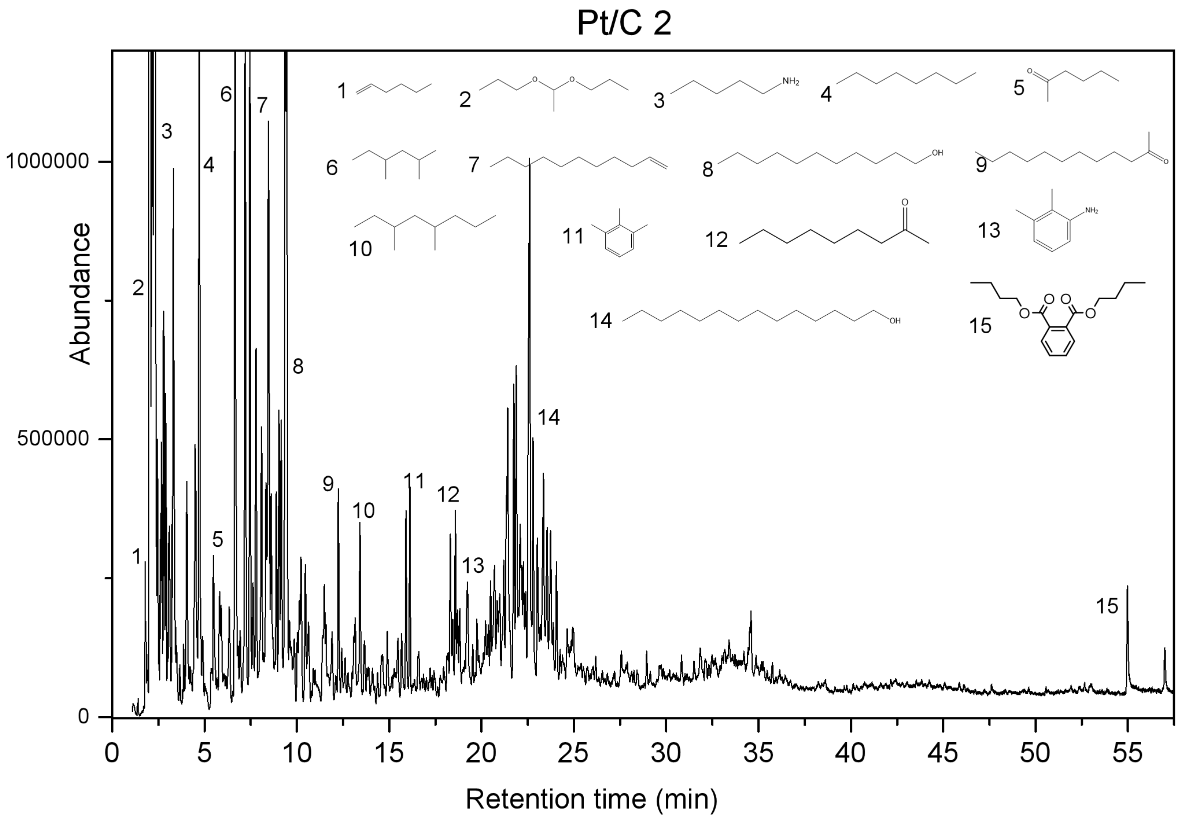

Fig. S 7 gc-ms analysis of liquid product using Pt/C 2 catalyst

Small molecule hydrocarbons and hydrocarbon derivatives: 2-methyl-1,3-butadiene (2-methly-1,3-butadiene), 2,2-dimethyl-3-heptene (2,2 -dimethly-3-heptene), nonane, 3-ethyl-hexane, 2,3-dimethyl-2-hydroxy-3-pentene (2,3- dimethyl-2-hydroxy-3-penten), 2-hexene.

Table S 3 Structure of small molecule hydrocarbons and derivatives in the liquid ph ase of sludge pyrolysis

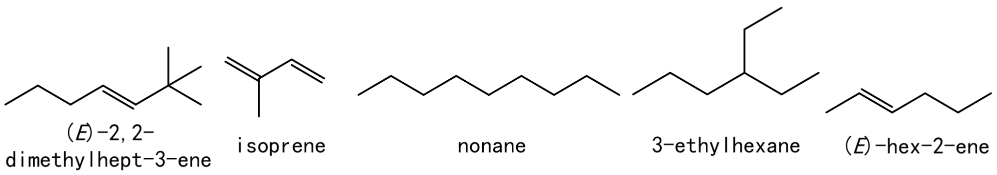

(*E*)-2, 2-dimethylhept-3-ene   isoprene   nonane   3-ethylhexane   (*E*)-hex-2-ene

Cycloaliphatic compounds: 1,2-dimethyl-cyclohexane, 1,2-cyclohexene, ethanone cyclohexene, ethylcyclohexane, 1,1-dimethyl 2,5-dimethyl-5-carbonyl-cyclohexen, bromo cyclohexane.

Table S 4 Structure of alicyclic compounds in the liquid phase of sludge pyrolysis

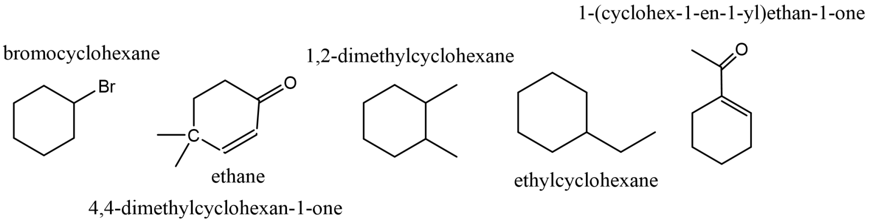

Aromatic compounds: Styrene, fluoromethyl-benzene, diacetylphenylhydrazine, (2-acetyl-1-phenylhydrazine), benzene, (2-dimethylpropyl)).

Table S 5 Structure of aromatic compounds in the liquid phase of sludge pyrolysis

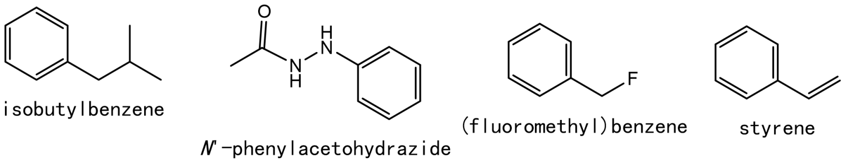

Heterocyclic compounds: pyridine, 4-ethyl, 1-oxide, pyridinone, 6-Hydroxy-pyridinone, 2-methly-furoate, 1-methyl-2-pyrrole formaldehyde (1H-pyrrole-2-carboxaldehyde, 1-methyl), trioxane, 2-methyl-aziridine, Pyrrolidine, 2,5-dimethylfuran.

Table S 6 Structure of heterocyclic compounds in sludge pyrolysis liquid phase

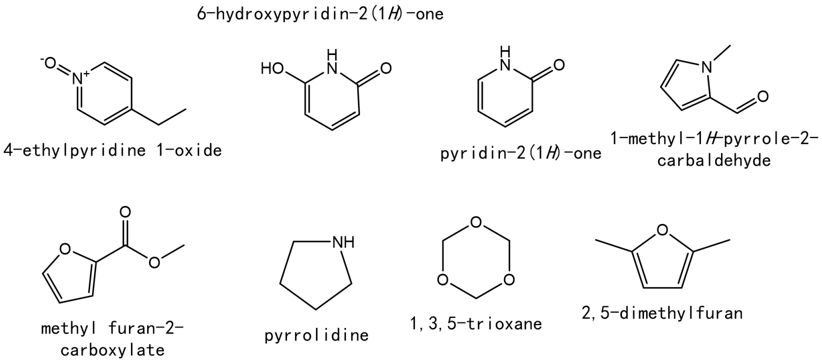

Small molecule esters: Bhylcycl opropane carboxylate, 1-methylethyl ester.

Table S 7 Structure of small molecule esters in the liquid phase of sludge pyrolysis

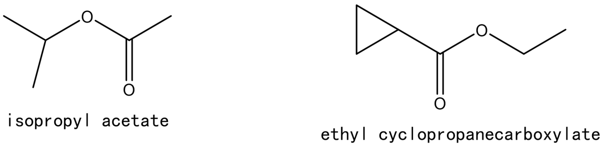

Small Molecule Ketones: Nonanone, 2,2-methlypropyl-3-hexanone, 3,5-dimethylcyclohexanone (3,5- dimethyl-cyclohexanone, 2-hexanone, 3-methylcyclopentanone, 3-hydroxy-3-methyl-2 -butanone).

Table S 8 Structure of small molecule ketones in the liquid phase of sludge pyrolys is

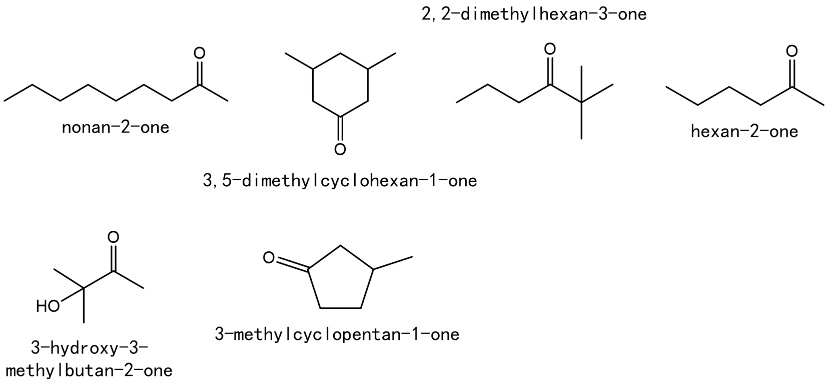

Small molecule alcohols: 3-methyl-1-butanol, 1,3-dichloro-2-propanol.

Table S 9 Structure of small molecule alcohols in the liquid phase of sludge pyroly sis

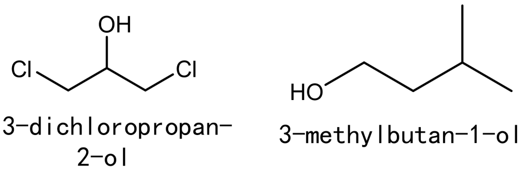

Supplement: Supplementary file 1 — Supplementary Information [file 41598_2020_75980_MOESM1_ESM.pdf]
